# Effects of Aging in Multisensory Integration: A Systematic Review

**DOI:** 10.3389/fnagi.2017.00080

**Published:** 2017-03-28

**Authors:** Alix L. de Dieuleveult, Petra C. Siemonsma, Jan B. F. van Erp, Anne-Marie Brouwer

**Affiliations:** ^1^Predictive Health Technologies, Netherlands Organisation for Applied Scientific ResearchLeiden, Netherlands; ^2^Perceptual and Cognitive Systems, Netherlands Organisation for Applied Scientific ResearchSoesterberg, Netherlands; ^3^Thim van der Laan, University for PhysiotherapyNieuwegein, Netherlands; ^4^Faculty of Health, University of Applied Sciences LeidenLeiden, Netherlands; ^5^Human Media Interaction, Electrical Engineering, Mathematics and Computer Science, University of TwenteEnschede, Netherlands

**Keywords:** aging, elderly, multisensory integration, multimodal, activities of daily living

## Abstract

Multisensory integration (MSI) is the integration by the brain of environmental information acquired through more than one sense. Accurate MSI has been shown to be a key component of successful aging and to be crucial for processes underlying activities of daily living (ADLs). Problems in MSI could prevent older adults (OA) to age in place and live independently. However, there is a need to know how to assess changes in MSI in individuals. This systematic review provides an overview of tests assessing the effect of age on MSI in the healthy elderly population (aged 60 years and older). A literature search was done in Scopus. Articles from the earliest records available to January 20, 2016, were eligible for inclusion if assessing effects of aging on MSI in the healthy elderly population compared to younger adults (YA). These articles were rated for risk of bias with the Newcastle-Ottawa quality assessment. Out of 307 identified research articles, 49 articles were included for final review, describing 69 tests. The review indicated that OA maximize the use of multiple sources of information in comparison to YA (20 studies). In tasks that require more cognitive function, or when participants need to adapt rapidly to a situation, or when a dual task is added to the experiment, OA have problems selecting and integrating information properly as compared to YA (19 studies). Additionally, irrelevant or wrong information (i.e., distractors) has a greater impact on OA than on YA (21 studies). OA failing to weigh sensory information properly, has not been described in previous reviews. Anatomical changes (i.e., reduction of brain volume and differences of brain areas’ recruitment) and information processing changes (i.e., general cognitive slowing, inverse effectiveness, larger time window of integration, deficits in attentional control and increased noise at baseline) can only partly explain the differences between OA and YA regarding MSI. Since we have an interest in successful aging and early detection of MSI issues in the elderly population, the identified tests form a good starting point to develop a clinically useful toolkit to assess MSI in healthy OA.

## Introduction

The growing interest in the mechanisms of aging is probably directly related to the increasing population of older adults (OA) in our society. Indeed, the world global life expectancy is increasing while the global world fertility has steadily declined (Crampton, 2009; World Health Organization, 2015). As a consequence, the world population of 60 years old people and older, considered as OA (World Health Organization, 2015), is expected to increase from 10.8% of the population in 2009 to 22% by 2050 (Crampton, 2009; World Health Organization, 2015). These are major changes that need to be studied in order to understand their impacts on our society, identify the emerging challenges (World Health Organization, 2015) and to assist this growing population in maintaining a high quality of life and independency.

A reduction in brain volume has been claimed to be the cause of major changes in OA’ abilities (Hedman et al., 2012). After the age of 35, this reduction accelerates progressively with age to an annual brain volume loss of 0.5% at age 60 (Hedman et al., 2012). Motor abilities (Newell et al., 2006; Van Houwelingen et al., 2014) and cognitive abilities (Glisky, 2007; Yaffe et al., 2009), have been studied to investigate age-related changes. In comparison to younger adults (YA), OA showed a decline in the range of movements, gait speed, attention, memory, perception, and decision making (Newell et al., 2006; Glisky, 2007; Yaffe et al., 2009; Van Houwelingen et al., 2014). OA also show more symmetrical activation of the brain compared to YA (Cabeza, 2002; Peters, 2006; Greenwood, 2007; Park and Reuter-Lorenz, 2009) and a dedifferentiation of the brain, OA recruit more areas and have a loss of specialization of the brain circuits for a task (Baltes et al., 1980; Cabeza, 2002).

To live independently and age successfully, an individual needs a sufficient level of mobility. This has been defined as “the ability to move one’s own body through space” (Lowry et al., 2012) and includes activities such as walking, reaching and climbing stairs. These activities are a pre-requisite to be able to perform the activities of daily living (ADLs; Lowry et al., 2012). ADLs encompass both basic activities of daily living (basic ADL) and instrumental activities of daily living (IADL). Basic ADL refers to people’s daily self-care activities, such as getting ready in the morning, get from place to place during the day, and going to bed in the evening (Wiener et al., 1990), for example, bathing, dressing, toileting, transferring, continence and feeding (Katz et al., 1963). IADL refers to activities that need more cognition and are essential to live independently within the community, such as the ability to use a phone or to do shopping (Lawton and Brody, 1969; Wiener et al., 1990; Mamikonian-Zarpas and Laganá, 2015). In this systematic review, we will focus on changes that have an impact on the motor performance of all three aspects of ADLs: mobility, basic ADLs and IADLs.

To perform ADLs, the brain’s ability to extract, organize and process information is called upon. Information is received through the senses, processed and associated with prior memories, experiences, and knowledge in order to produce a focused response, this phenomenon is known as sensory integration (Lipsitz, 2002; Freiherr et al., 2013; Carriot et al., 2015). The integration of multiple unisensory signals from the environment which need to be combined into a unique and coherent percept is known as multisensory integration (MSI; Stein and Meredith, 1990; Freiherr et al., 2013; Mudrik et al., 2014; Bolognini et al., 2015; Talsma, 2015). Different brain areas have been shown to be involved in the process of MSI, and particularly the superior temporal sulcus (STS; Calvert and Thesen, 2004; Clemo et al., 2012). This part of the brain is located in the temporal lobe, one of the regions primarily affected by brain volume loss associated with aging (Peters, 2006). Accurate MSI is crucial for perception, cognitive processing and control of action (Stein and Meredith, 1990; Freiherr et al., 2013), processes that are essential for mobility, ADLs’ performance and to a greater extent, to live independently (Freiherr et al., 2013; Chiba et al., 2016). As a consequence, problems in MSI processes could lead to restrictions in performing ADLs and prevent elderly people to age in place and independently.

Single senses and body functions are known to deteriorate with aging, such as vision (Owsley, 2011), joint mobility (Yeh et al., 2015), muscle force (Cruz-Jentoft et al., 2010) and balance (Teasdale et al., 1991; Bugnariu and Fung, 2007). However, as far as we know, the impact of changes in MSI on age-related deterioration in ADLs is less well researched. The changes in performance in MSI tasks may be a more sensitive and earlier predictor for future ADL deterioration than in unisensory tasks. Therefore, the aims of this systematic review are to give an overview of measures that have been used to compare MSI between the healthy elderly population and YA and to summarize the results of these studies to see the effect of aging on MSI. Our future aim is to use the results found in this systematic review to develop a clinically useful toolkit for assessing the extent of MSI in healthy older individuals.

## Materials and Methods

This systematic review was written using the Preferred Reporting Items for Systematic Reviews and Meta-Analyses (PRISMA) statement (Moher et al., 2009). PRISMA is a 27-item checklist that aims to improve the reporting of systematic reviews and meta-analysis (Moher et al., 2009). The protocol of this systematic review has been registered in the PROSPERO database, international prospective register for systematic reviews under the registration number CRD42016036946^1^.

### Participants

The target population of this systematic review is the healthy elderly population of 60 years old and above. Since OA are likely to experience decline in functions, may have some limitations, or develop chronic diseases during their life, healthy OA were defined as OA not primarily labeled as having a disease. A comparison group of younger participants was included in order to investigate the effects of aging or a single group of participants including a range of participants from young to older individuals. The younger participants should be healthy, i.e., no current acute, severe or chronic disease.

### ADLs Selection

We focus on changes that have an impact on the motor performance of all three aspects of ADLs: mobility, basic ADLs and IADLs. We are primarily interested by activities or senses that are crucial to perform ADLs such as vision or balance, therefore, we decided to not include tests on speech (although needed for interaction with others, speech in itself is not essential for performance of ADLs), emotion perception, taste, olfaction and semantic processes.

### Study Selection

**Figure 1:**
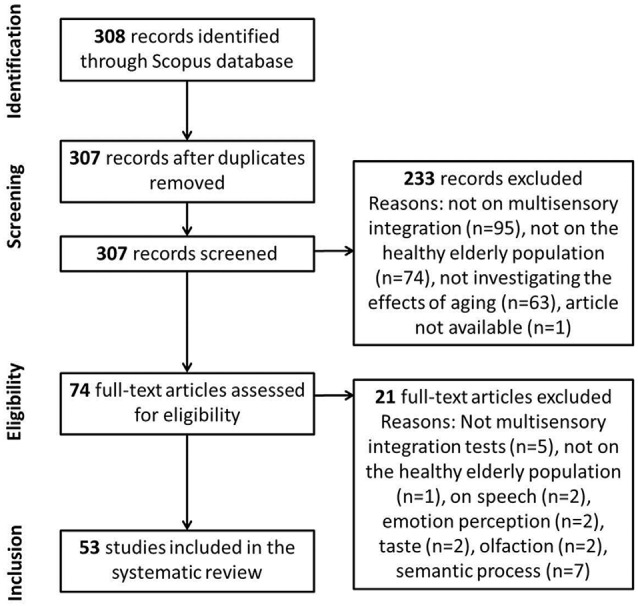
**Flow of information through the different phases of the systematic review**.

The systematic review contains four selection phases (see Figure 1), as suggested by PRISMA. The first phase is the identification of the records through database searching. The second phase is the screening of the records. During this phase, duplicates are removed and records are checked for the selection criteria. The third phase is the eligibility phase, where the full-text articles are rated for eligibility criteria, and finally, in the inclusion phase, suitable articles are included in the systematic review.

The identification phase was performed in Scopus, an abstract and indexing database with full-text links produced by the Elsevier Co. (Burnham, 2006). It is the largest abstract and citation database of peer-reviewed literature dating back to 1970 (Elsevier, 2016). This database covers 100% of MEDLINE, 100% of EMBASE and 100% of Compendex (Burnham, 2006).

Articles were included if they investigated an effect of aging on MSI in the healthy elderly population. Records were searched from the earliest records available to January 20, 2016. The search strategy was developed reading relevant reviews and articles on MSI. Keywords found in these articles were adapted to be used in Scopus. The keywords used for the search in Scopus are detailed in the Table 1. Limits were set to restrict the search results to elderly humans and to the document type (articles). Finally, we excluded studies focusing on speech, emotion perception, taste, olfaction, semantic processes and studies concerning several common diseases in the elderly population (see Table 1). Three hundred and eight articles were found with the combination of these criteria.

**Table 1:** **Table of the research strategy done in the Scopus database to find tests of multisensory integration in the healthy elderly population**.

Scopus	Query	Research in:	Items found
**#1**	“Sensory integration” OR “multisensory integration” OR “crossmodal integration” OR “cross-modal integration” OR “intersensory integration” OR “multimodal integration” OR “crossmodal illusion*” OR “cross-modal illusion*” OR multisensory OR crossmodal OR cross-modal OR “crossmodal sensory integration” OR “cross-modal sensory integration” OR “multisensory interaction*”	Article Title, Abstract, Keywords	11,005
**#2**	Measurement* OR Test* OR performance OR assessment* OR “Test development” OR “task performance” OR “disability evaluation” OR “Feasibility studies” OR validity OR reliability OR study* OR results*	Article Title, Abstract, Keywords	14,411,633
**#3**	Combine #1 AND #2		5127
**#4**	Limit to (Humans OR human) AND (Limit to (DOCTYPE, article))		3241
**#5**	Limit to (“aged”, “aging”)		394
**#6**	Exclude (“Speech perception”, “Speech Perception”, “Speech”)		351
**#7**	Exclude (“Alzheimer Disease”, “Alzheimer disease”, “Parkinson Disease”, “Parkinson disease”, “Aphasia”, “Dementia”, “Disease severity”, “Brain damage”, “Brain injury”, “Stroke”, “Neglect”, “Brain damage, chronic”, “Cerebrovascular accident”, “Cognition disorders”, “Neurologic disease”, “Schizophrenia”)		308

*replace multiple characters anywhere in a word. Example: behav* finds behave, behavior, behaviour, behavioural, behaviourism, etc. (http://help.elsevier.com/app/answers/detail/a_id/2950/p/8150).

During the screening phase, duplicates were removed (*n* = 1) and records were checked for the selection criteria to include MSI, healthy elderly population (60 years old and older) and investigation of the aging effects in the multisensory task. This resulted in the inclusion of 74 out of the 308 articles for further assessment in the eligibility phase. Criteria for eligibility were: measurement of MSI in the elderly population and a comparison group of YA. Articles on speech that were still in the resulting articles, on emotion perception, taste, olfaction and semantic processes were also excluded from the systematic. Finally, 53 studies were included in the systematic review (see Figure 1).

### Quality Assessment

All 53 studies were rated for quality to evaluate the risks of bias in the results (see Appendix 1 in Supplementary Material). The Newcastle-Ottawa quality assessment scale (NOS) was used to rate the articles (Table 3 in Appendix 1 in Supplementary Material; Wells et al., 2012). The NOS assessment was designed to rate nonrandomized studies, including case-control and cohort studies and consists of eight items grouped into three sections: selection, comparability and exposure. Each item was rated for a maximum score of one star. The maximum summed score was eight stars. In line with other systematic reviews (Qi et al., 2015; Zhang et al., 2015; Ma et al., 2016) we used five stars out of eight as cut off in this systematic review. The studies that failed to reach five stars in the NOS were excluded from the summary of the results (four studies).

### Groups

The resulting studies were grouped according to the specific combination of modalities that were tested. The studies were described for their key study characteristics: Title, first author, year of publication, participants recruited, material used, experiments done and the results found of aging (see Appendix 2 in Supplementary Material).

### Analysis of the Results

For each group of modalities, articles were sorted by type of test performed (for example detection tasks, temporal order judgment tasks or sound-induced flash illusion tasks). The results of aging for each type of test were summarized for each group.

## Results

### Quality Assessment

According to the NOS (Table 3 in Appendix 1 in Supplementary Material; Wells et al., 2012), most of the studies included in this systematic review show good scores of quality (*n* = 49). Only four studies have been rated less than five stars out of eight (Woollacott et al., 1987; Prioli et al., 2005; Chan et al., 2014b; Cohen et al., 2014) and were excluded from the analysis of the results.

### Groups of Modalities

The groups of modalities found according to the specific combination of modalities that were tested were the following: visual and auditory modalities tests (*n* = 22 articles including a total of 32 tests), visual, vestibular and somatosensory modalities tests (*n* = 13 articles including a total of 20 tests), visual and somatosensory modalities tests (*n* = 8 articles including a total of 11 tests) and other modalities (*n* = 6 articles including a total of 6 tests).

### Participants

Almost all the studies included in the systematic review investigated the effects of aging by comparing the response of a group of OA to a group of YA (sometimes with other groups of participants as well). Only two studies explored the effects of aging within one group of participants, Cham et al. (2007) with a group from 41 to 83 years old participants (mean age 65) and Strupp et al. (1999) with a group from 21 to 81 years old participants (mean age 46). The group size and age range per groups of modalities for the other 47 studies are summarized below in Table 2. In most of the studies (*n* = 44), the group of YA was a control of the OA group for additional factors to unsure that the groups are comparable: gender, education, intelligence and/or level of cognition.

**Table 2:** **Size and age range of the different groups of modalities**.

Group of modalities	Group size (range number of participants, mean number)		Age range of the group (years)	
	OA	YA	OA	YA
Visual and auditory	8–30, 18	6–30, 18	60–89	18–41
Visual, vestibular and somatosensory	7–48, 17	7–24, 15	60–85	18–65
Visual and somatosensory	12–30, 20	9–30, 18	60–92	16–37
Other	10–20, 16	10–20, 15	61–85	16–37

### Summary of the Results on Aging

A description of key features and results for the individual articles are presented in Appendix 2 in Supplementary Material.

#### Tests on the Visual and Auditory Modalities

##### Types of visual and auditory tests

Tests on the visual and auditory modalities (*n* = 32 tests) explored vision and audition based on participants’ reaction times when responding to unimodal or bimodal stimuli to investigate their impact on MSI compared to unisensory performance. Distractors have been added to some experiments. Several types of tests were used in these experiments. Some authors used a simple unimodal or bimodal detection task (Townsend et al., 2006; Peiffer et al., 2007; Hugenschmidt et al., 2009a). Other authors investigated reaction times during unimodal or bimodal localization tasks (Hugenschmidt et al., 2009b; Campbell et al., 2010; Stephen et al., 2010; Dobreva et al., 2012; Wu et al., 2012) with spatial cueing (Guerreiro et al., 2012) or using peripheral vision (Cui et al., 2010; Dobreva et al., 2012) or the ability to remember or localize a stimulus in one modality while ignoring another modality (Diederich et al., 2008; Guerreiro et al., 2014, 2015). Other authors used judgment tasks; audiovisual temporal order judgment task (Setti et al., 2011b; de Boer-Schellekens and Vroomen, 2013; Fiacconi et al., 2013), audiovisual asynchrony judgment (Chan et al., 2014a) or audiovisual *n*-back task (Guerreiro and Van Gerven, 2011; Guerreiro et al., 2013). Finally, in some articles, participants had to perform a sound-induced flash illusion task (Setti et al., 2011a; DeLoss et al., 2013; McGovern et al., 2014).

##### Findings on the visual and auditory modalities

Three main findings emerged from the results of the experiments (for details see Appendix 2 in Supplementary Material).

First, OA seemed to integrate more multisensory (audiovisual) information compared to YA. In other words: OA used all audiovisual information present in the environment (Townsend et al., 2006; Peiffer et al., 2007; Diederich et al., 2008; Hugenschmidt et al., 2009b; Stephen et al., 2010; Guerreiro et al., 2012, 2014, 2015; Wu et al., 2012; DeLoss et al., 2013). Both groups showed better performance in multisensory tasks compared to unimodal tasks but OA seemed to benefit more from enriched multisensory information than YA (Diederich et al., 2008; Hugenschmidt et al., 2009b; de Boer-Schellekens and Vroomen, 2013; DeLoss et al., 2013; Guerreiro et al., 2014, 2015). When performing detection tasks, OA showed similar responses to MSI as YA (Townsend et al., 2006; Hugenschmidt et al., 2009a,b; Guerreiro et al., 2012, 2014, 2015; Fiacconi et al., 2013) or even faster responses to multisensory information compared to YA (Peiffer et al., 2007). However, OA were still impaired at performing correctly in the task compared to YA. They needed more time to perform accurately in selective attention tasks compared to YA (Diederich et al., 2008; Hugenschmidt et al., 2009b; Stephen et al., 2010; DeLoss et al., 2013; Guerreiro et al., 2014, 2015) and were less accurate at localizing a target in space or detecting asynchrony compared to YA (Stephen et al., 2010; Dobreva et al., 2012; Wu et al., 2012). The effects of age on audiovisual temporal order judgment were not clear. Some authors found a decline of sensitivity in this task from 50 years of age (de Boer-Schellekens and Vroomen, 2013), others found no age-related differences in this task (Fiacconi et al., 2013) and other authors found increased age-related differences (Setti et al., 2011b). Furthermore, de Boer-Schellekens and Vroomen (2013) showed that additional noise compensated the loss of sensitivity that they found, particularly in OA.

Second, distractors or inaccurate information (e.g., visual bias) tended to have a greater influence on the performance of OA compared to YA, thus OA had more trouble ignoring irrelevant information (Hugenschmidt et al., 2009a; Guerreiro and Van Gerven, 2011; Dobreva et al., 2012; Wu et al., 2012; DeLoss et al., 2013; Guerreiro et al., 2013; McGovern et al., 2014).

Third, a broader time window of audiovisual integration was found in OA compared to YA (Peiffer et al., 2007; Diederich et al., 2008; Wu et al., 2012). The time window of integration is the time period for possible integration. A first stimulus “opens the window” and, to be integrated, a second stimulus must happen inside this time window (Colonius and Diederich, 2004a).

#### Tests on the Visual, Vestibular and Somatosensory Modalities

##### Types of visual, vestibular and somatosensory tests

Tests on the visual, vestibular and somatosensory modalities (*n* = 20 tests) investigated the combination of modalities while disturbing the sensory inputs, for example by introducing a perturbation or introducing wrong information that needs to be ignored in order to perform the task accurately. According to the authors, this assists in identifying the modalities that are preferentially used by the participants and how accurately they use the information available. Only one study did not perturbed any sensory input to look at the differences between OA and YA (Hugenschmidt et al., 2014). *Visual inputs* have been perturbed in different ways. First of all, visual input has been suppressed by some authors by asking participants to simply close their eyes (Stelmach et al., 1989; Teasdale et al., 1991; Cham et al., 2007; Bellomo et al., 2009). Other authors limited the visual input using active shutter googles (Allison et al., 2006; Eikema et al., 2014) or blurry vision (Deshpande and Patla, 2007). Others used optic flows to introduce a visual movement while participants were performing a task (Allison et al., 2006; Eikema et al., 2014). Finally, some authors introduced conflicting visual inputs that were not consistent with the information from the other modalities, such as a sway-referenced visual scene (Redfern et al., 2001, 2009; Allison et al., 2006; Cham et al., 2007) or an optic flow with the center of expansion gradually deviating to the left or to the right while subjects had to walk straight (Berard et al., 2012).

*Somatosensory inputs* have been perturbed, using devices such as a compliant surface (Deshpande and Zhang, 2014), bilateral Achilles tendon vibration (Eikema et al., 2014), and a movable touch plate where the fingertip is placed (Allison et al., 2006). Most authors used a movable platform or a moving room to produce a referenced sway to the floor (Stelmach et al., 1989; Redfern et al., 2001, 2009; Allison et al., 2006; Cham et al., 2007).

*Vestibular inputs* have been perturbed using galvanic vestibular stimulation (GVS; Deshpande and Patla, 2007; Deshpande and Zhang, 2014; Eikema et al., 2014) or a rotatory chair (Bates and Wolbers, 2014).

##### Findings on the visual, vestibular and somatosensory modalities

Four main findings can be summarized from the results of the experiments (for details see Appendix 2 in Supplementary Material).

First, both groups of participants showed better performance in navigation tasks when more information was available in the environment (Deshpande and Patla, 2007; Redfern et al., 2009; Berard et al., 2012; Bates and Wolbers, 2014; Deshpande and Zhang, 2014; Eikema et al., 2014). However, OA showed a poorer and more variable performance in navigation tasks compared to YA, even if their performance was improved under multisensory conditions (Deshpande and Patla, 2007; Redfern et al., 2009; Berard et al., 2012; Bates and Wolbers, 2014; Deshpande and Zhang, 2014; Eikema et al., 2014; Hugenschmidt et al., 2014).

Second, the perturbations of modalities and dual task-conditions led to an increase of body sway dispersion in all groups, but this effect was larger in OA, leading to more losses of balance (Stelmach et al., 1989; Teasdale et al., 1991; Deshpande and Patla, 2007; Redfern et al., 2009; Berard et al., 2012). This effect on body sway was even larger when more than one modality was disrupted or when a dual task was added (Redfern et al., 2001, 2009; Deshpande and Patla, 2007; Deshpande and Zhang, 2014; Eikema et al., 2014).

Third, when the accuracy of modalities was restored, OA failed to weigh and use properly the accurate information. This results in OA being even more perturbed, while the YA adapted rapidly (Teasdale et al., 1991; Berard et al., 2012; Eikema et al., 2014).

Fourth, OA relied less than predicted, by Bayesian models, on visual landmarks in a navigation task when they needed to find the right direction in a room to reach a specific location (Bates and Wolbers, 2014). This failure of using information was also seen when GVS was added to help the subjects reduce postural sway (Eikema et al., 2014), OA were unable to properly use the information.

#### Tests on the Visual and Somatosensory Modalities

##### Types of visual and somatosensory tests

Tests on the visual and somatosensory modalities (*n* = 11 tests) investigated the degree to which participants can recognize the same object in two different modalities. Others investigated the visual and somatosensory modalities based on participants’ reaction times when responding to unimodal or bimodal stimuli to investigate the impact of MSI compared to unisensory integration. Perturbations of the modalities inputs were added to some experiments (*n* = 4 tests).

Some authors tested visual-to-tactual recognition or tactual-to-visual recognition (Oscar-Berman et al., 1990; Norman et al., 2006), others did temporal order judgment tasks with or without distractors in the same or other modality (Poliakoff et al., 2006a,b), others did the Fitts’ task (Temprado et al., 2013) and a tactual transfer task (Coté and Schaefer, 1981). Other authors looked at the effects of the suppression or disturbance of the input of a modality; Brodoehl et al. (2015) investigated the changes in somatosensory detection threshold when participants opened and closed their eyes; Strupp et al. (1999) investigated the effects of somatosensory perturbation using dorsal muscle vibration on the performance in a task where participants were asked to move a laser spot to the position they perceived as straight ahead.

##### Findings on the visual and somatosensory modalities

Two main findings can be summarized from the results of the experiments (for details see Appendix 2 in Supplementary Material).

First, it seems that aging affected cross modal (visual-somatosensory) shape discrimination but not unimodal discrimination. OA needed more time to accurately perform the two kinds of task compared to YA as well as to perform properly the Fitts’ task.

Second, OA seemed to be more affected in their performance by visual and somatosensory distractors or perturbations compared to YA.

#### Tests on Other Combinations of Modalities

##### Types of tests on other combinations of modalities

Tests on the visual, auditory and somatosensory modalities (*n* = 2 tests) explored the effects of orienting and alerting through unimodal and multimodal cues in reaction time tasks. This was done to assess the effectiveness of the different unisensory and multisensory cues. Mahoney et al. (2011, 2012) tested the multisensory facilitation of multisensory information as compared to unisensory information in a simple reaction task. They also tested the effects of orienting and alerting unimodal and multimodal cues in a forced-choice reaction time task.

Test on the auditory and somatosensory modalities (*n* = 1 test) investigated the capacity of the participants to follow with finger tapping a metronome presented unimodally or bimodally, again to explore the differences between unisensory and MSI (Elliott et al., 2011).

Test on the visual, auditory and vestibular modalities (*n* = 1 test) studied the reaction time of the participants after the visual and vestibular inputs were perturbed separately or simultaneously. The authors identified the modalities that were preferentially used by the subjects and how they used the information available. Furman et al. (2003) did three different tasks, a simple reaction time task, a disjunctive reaction time task and a forced-choice reaction time task done while participants were sitting on a rotational chair with vision only, vestibular only or both.

Tests on the auditory, somatosensory and vestibular modalities (*n* = 2 tests) explored the control of posture while participants performed a dual task. These tests enabled the authors to explore the effects of an attention task on the integration of sensory inputs. Mahboobin et al. (2007) used a posture platform to assess postural control of the participants while doing an auditory choice reaction time task or an auditory vigilance task.

##### Findings on other combinations of modalities

Two main findings can be summarized from the results of the experiments (for details see Appendix 2 in Supplementary Material).

First, both groups showed faster and more accurate responses under multisensory conditions than under unisensory conditions and in every experiment, OA showed longer RT compared to YA (Furman et al., 2003; Elliott et al., 2011; Mahoney et al., 2011, 2012; Bisson et al., 2014). The multisensory facilitation seemed to be modality specific depending on age group; OA showed a greater RT benefit when processing visual-somatosensory information while YA showed greater benefits from audiovisual and audio-somatosensory information (Mahoney et al., 2011). OA seemed to benefit more from audiovisual orienting cues and YA seemed to benefit more from audio-somatosensory orienting cues compared to other unisensory or multisensory cues (Mahoney et al., 2012).

Second, modality perturbations (e.g., temporal irregularity of the auditory metronome) or the addition of a dual task led to a degradation of task performance in both groups of subjects, but this effect was larger in OA (Mahboobin et al., 2007; Elliott et al., 2011; Bisson et al., 2014).

## Summary of Findings

Below we summarize the main findings of our literature study.

### OA Maximize MSI

The studies included in this review show that OA rely more on all their senses compared to YA. OA benefit more from multisensory enrichment in the environment. They use all information available to them to perform a task and benefit more from bimodal stimuli compared to unimodal stimuli (Furman et al., 2003; Townsend et al., 2006; Deshpande and Patla, 2007; Peiffer et al., 2007; Diederich et al., 2008; Hugenschmidt et al., 2009b; Redfern et al., 2009; Stephen et al., 2010; Elliott et al., 2011; Mahoney et al., 2011, 2012; Berard et al., 2012; Guerreiro et al., 2012, 2014, 2015; Wu et al., 2012; de Boer-Schellekens and Vroomen, 2013; DeLoss et al., 2013; Bates and Wolbers, 2014; Deshpande and Zhang, 2014; Eikema et al., 2014). They also demonstrate a broader time window of integration compared to YA (Peiffer et al., 2007; Diederich et al., 2008; Wu et al., 2012), meaning that the time period used by OA to integrate information from different senses as a unique multisensory percept is larger compared to YA. This gives OA the opportunity to integrate more multisensory information. Furthermore, OA show the same or faster responses to multisensory information than YA during selective attention tasks (Townsend et al., 2006; Peiffer et al., 2007; Hugenschmidt et al., 2009a,b; Guerreiro et al., 2012, 2014, 2015; Fiacconi et al., 2013). These results suggest that selective attention remains intact in the elderly population in simple cases and that MSI can help driving attention particularly for elderly people. All these results show that OA maximize the use of MSI by taking into account every information of the environment.

#### OA’ Performance in the Tasks is Impaired Compared to YA

Despite the shown enhanced use of MSI and intact selective attention, OA perform less well than YA in tasks that require more cognitive function than simple stimulus detection tasks. OA need more time to accurately perform more complex tasks in comparison to YA and show longer reaction times (e.g., selective attention task or space localization task; Furman et al., 2003; Diederich et al., 2008; Hugenschmidt et al., 2009b; Stephen et al., 2010; Elliott et al., 2011; Mahoney et al., 2011, 2012; Dobreva et al., 2012; Wu et al., 2012; DeLoss et al., 2013; Temprado et al., 2013; Guerreiro et al., 2014, 2015). In addition, OA are less accurate and more variable at performing tasks like navigation or localizing a target in space (Deshpande and Patla, 2007; Redfern et al., 2009; Berard et al., 2012; Bates and Wolbers, 2014; Deshpande and Zhang, 2014; Eikema et al., 2014; Hugenschmidt et al., 2014).

#### OA are Impaired in Properly Weighing Sensory Information

Additionally, OA were found to be impaired in properly weighing relevant and irrelevant sensory information from one’s own body and from the environment. Specifically, data suggest that, in comparison to YA, they do not properly adjust information that is unreliable (disrupted or taken away) or non-informative (distractors). They continue to use all environmental information when they should not (Stelmach et al., 1989; Teasdale et al., 1991; Strupp et al., 1999; Redfern et al., 2001, 2009; Allison et al., 2006; Poliakoff et al., 2006a; Deshpande and Patla, 2007; Hugenschmidt et al., 2009a; Elliott et al., 2011; Guerreiro and Van Gerven, 2011; Berard et al., 2012; Dobreva et al., 2012; Wu et al., 2012; DeLoss et al., 2013; Guerreiro et al., 2013; Bisson et al., 2014; Deshpande and Zhang, 2014; Eikema et al., 2014; McGovern et al., 2014; Brodoehl et al., 2015). These results were seen in several tests assessing integration of all combinations of modalities found in this review. These tests include the sound-induced flash illusion, n-back tasks with distractors, walking and navigation tasks, postural tasks with or without cognitive dual task, selective attention task with distractors, visual straight ahead tasks, control of movement timing tasks and visual-vestibular task with cognitive dual task. When the accuracy of a modality was restored in the trials, OA failed to use the correct information properly and as a result, they were even more perturbed while the YA adapted rapidly (Teasdale et al., 1991; Berard et al., 2012; Eikema et al., 2014). They are thus impaired in rapidly adapting their behavior to the environment which can be an issue for the performance of ADLs. As far as we know, the fact that OA are impaired at properly weighing sensory information has not been described earlier in the literature.

#### A Dual Task Decreases Task Performance

Dual tasking involves the concurrence of two different activities and requires high attentional demand. The results show that the addition of a dual task decreases the performance of both age groups, and that this effect is larger in the elderly population (Redfern et al., 2001, 2009; Mahboobin et al., 2007; Bisson et al., 2014). It seems that OA are unable to compensate for the increase in attentional demands and have difficulties to accurately perform multiple tasks at the same time.

## Discussion of Findings

Together, all of these results point at an inclination of OA to integrate all information available to them in the environment while YA tend to weigh information present in the environment in order to use the relevant ones. In the following, views and theories that have been put forwards in the literature to explain MSI differences between OA and YA will be discussed: (1) anatomical differences; (2) information processing differences; and (3) the view that OA have trouble to weigh sensory information. Finally, speculations on the potential causes of this age-related change will be described.

### Anatomical View

This view has two parts: the reduction of brain volume and the differences in brain recruitment strategies which reveal anatomical differences between OA and YA and could be a part in the explanation of the differences found between these two groups of participants regarding MSI.

#### Reduction of Brain Volume

A volume reduction of the temporal lobe has been hypothesized to be an anatomical cause of the changes in MSI found in the elderly population. Several brain areas have been found to contribute to the process of MSI, the impact of one sensory modality on the brain activity produced by another sensory modality. The STS and the superior colliculus (SC) have been found to be major actors of this process (Calvert and Thesen, 2004; Clemo et al., 2012). The STS and the SC have been shown to receive projections from areas involved in visual processes, auditory processes, and somatosensory processes (Clemo et al., 2012). Other regions of the brain have been found to be involved in multisensory processing, for instance, the claustrum, the suprageniculate and medial pulvinar nuclei of the thalamus and the amygdaloid complex (Calvert and Thesen, 2004).

After 35 years of age, brain volume starts to reduce (Hedman et al., 2012). While several parts of the brain are affected by this volume loss, the prefrontal cortex and the striatum are the most affected (Peters, 2006). The volumes of the temporal lobe, cerebellar vermis, cerebellar hemispheres and hippocampus are also decreased by age as well as the prefrontal white matter (Peters, 2006). The STS, which is highly involved in MSI processes as seen above, is situated in the temporal lobe of the brain.

The reduction of brain volume observed in the elderly population has been claimed to be the cause of major changes in OA’ capacities (Hedman et al., 2012) and changes on brain activation (Peters, 2006).

#### Brain Recruitment Strategies

It has been shown that brains of OA tend to show more symmetrical activation than younger brains (Cabeza, 2002; Peters, 2006; Greenwood, 2007; Park and Reuter-Lorenz, 2009). This hemispheric asymmetry reduction in OA is called HAROLD (Cabeza, 2002). Different explanations have been explored to explain these findings. A failure to recruit the specific areas needed for the task and inhibition of the non-relevant areas, an attenuation of the response seen in YA or a compensation strategy of the aging process have been proposed (Peters, 2006; Park and Reuter-Lorenz, 2009). HAROLD was found to be correlated with higher performances in task execution in the elderly population, leading to the hypothesis that these changes occur to preserve the good functioning of cognition in OA (Greenwood, 2007; Park and Reuter-Lorenz, 2009). Additionally, during multisensory tasks, OA were shown to recruit more brain areas than YA (Townsend et al., 2006; Heuninckx et al., 2008; Venkatraman et al., 2010).

These changes of brain areas recruitment during MSI could serve as a compensation strategy for age-related deteriorations in individual sensory and motor systems and permit the elderly population to detect the stimuli as accurately as the YA (Cabeza, 2002). A dedifferentiation effect has also been proposed as an explanation (Baltes et al., 1980; Cabeza, 2002). Learning causes localized changes in specific areas of the brain needed for the task (Baltes et al., 1980; Bransford et al., 2000; Greenwood, 2007; Lövdén et al., 2013). Initially, several brain areas are recruited but as soon as the participant becomes an expert in the task, the expansion is followed by a renormalization of the activation map in which the most efficient circuits are selected (Lövdén et al., 2013). In the elderly population, this differentiation and specialization could be lost and OA start recruiting again a higher number of brain areas, the MSI control is unlearned (Baltes et al., 1980; Cabeza, 2002).

These anatomical modifications could be part of the changes that occur with aging regarding MSI by modifying the information processing in the brain of OA compared to YA. Although evidences of a link between anatomical changes and cognitive function have been described in the literature (Glisky, 2007), the exact nature of this relationship is not yet known and complex to investigate (Glisky, 2007).

### Information Processing View

This view describes the affected sensory integration of OA compared to YA and five hypotheses found in the literature attempting to explain these differences: the general cognitive slowing, the inverse effectiveness, a larger time window of integration, deficits in attentional control and the increased noise at baseline.

#### Affected Sensory Integration

MSI involves both top–down and bottom–up processes (Guerreiro et al., 2010; Talsma, 2015). MSI occurs pre-attentively in an automatic bottom-up process and is driven by the stimulus salience (Guerreiro et al., 2010; Talsma, 2015). The control of MSI is a top-down process driven by several components, expectations and goals for instance (Guerreiro et al., 2010; Talsma, 2015). However, an object integrated by more than one sensory system captures one’s attention more efficiently and proves that bottom-up integration can “drive” attention (Talsma, 2015). Additionally, the integration of stimuli depends on its relevance, for instance, a task-irrelevant sound associated with an attended visual stimuli will be more likely to be integrated compared to a task-irrelevant sound associated with an unattended visual stimulus (Guerreiro et al., 2010; van Erp et al., 2013; Talsma, 2015). Similar effects are found for visual and tactile stimuli (Philippi et al., 2008; Werkhoven et al., 2009; van Erp et al., 2014). These results show that top-down and bottom-up multisensory processes are closely interlinked. This systematic review shows that both top-down and bottom-up processes of MSI are affected by age. OA fail to use properly the bottom-up multisensory process of weighing information using their salience. Selective integration (top-down) is also hampered with age. As seen in the results above, OA are more affected than YA by dual tasks and especially cognitive tasks (Redfern et al., 2001, 2009; Mahboobin et al., 2007; Bisson et al., 2014).

Different theories have been explored to explain the results found in this systematic review in other reviews, particularly the increased use of MSI in the elderly population (Mozolic et al., 2012; Freiherr et al., 2013). These theories are explained below.

#### General Cognitive Slowing

OA were usually slower and impaired in task performance, particularly when the task was cognitively demanding or more difficult (Furman et al., 2003; Diederich et al., 2008; Hugenschmidt et al., 2009b; Stephen et al., 2010; Elliott et al., 2011; Mahoney et al., 2011, 2012; Dobreva et al., 2012; Wu et al., 2012; DeLoss et al., 2013; Guerreiro et al., 2014, 2015). Mozolic et al. (2012) argued that a unisensory presentation of a stimulus is a more demanding task than the multisensory presentation of this stimulus because the multisensory task provides redundant information (same stimuli in different modalities).

It could then be assumed that the high multisensory gain shown in the elderly population would be caused by MSI being a less demanding task than using unisensory information (Mozolic et al., 2012). However, when general cognitive slowing was reduced by the use of a simple task such as an audiovisual detection task (Peiffer et al., 2007), the higher MSI gain was still visible in OA compared to YA. Thus, general cognitive slowing cannot explain by itself the differences in multisensory processing between OA and YA.

#### Inverse Effectiveness

The inverse effectiveness is the principle that “decreasing the effectiveness of individual sensory stimuli increases the magnitude of multisensory enhancements” (Mozolic et al., 2012). It means that multisensory stimuli presented at a low level of salience (less intense or weak and ambiguous) are more likely to be integrated than unisensory stimulus presented at a high level of salience (Freiherr et al., 2013).

It is known that OA experience a functional decline in individual sensory systems (Teasdale et al., 1991; Bugnariu and Fung, 2007; Cruz-Jentoft et al., 2010; Owsley, 2011; Yeh et al., 2015). According to this principle of inverse effectiveness, this could lead to an increased multisensory benefit. However, in some studies included in this review, OA showed the same reaction times as the YA for unisensory stimuli (Townsend et al., 2006; Peiffer et al., 2007; Hugenschmidt et al., 2009a, b; Guerreiro et al., 2012, 2014, 2015; Fiacconi et al., 2013). This means that for some tasks, OA didn’t experience a functional decline effect in individual sensory systems compared to YA but still showed a multisensory facilitation. As a consequence, the inverse effectiveness cannot be the only process involved in the multisensory enhancement shown in the elderly population.

#### Larger Time Window of Integration

OA were found to have a “larger period for potential interaction” compared to YA as a consequence of broader distribution and increased response times (Peiffer et al., 2007; Diederich et al., 2008; Mozolic et al., 2012).

However, despite this larger time window of integration, increased reaction times and increased response variability actually reduce the probability of the overlapping of stimuli from different modalities in this time window (Diederich et al., 2008; Freiherr et al., 2013). Therefore, this hypothesis cannot explain why the use of MSI is higher in OA compared to YA (Mozolic et al., 2012; Freiherr et al., 2013).

#### Deficits in Attentional Control

Selective attention is the ability to focus on one stimulus or one modality while ignoring others (Mozolic et al., 2012; Freiherr et al., 2013). The brain activity of OA during selective attention for MSI has been shown to be different than the one of YA, who have an increased brain activity in areas associated with the attended modality and decreased brain activity in areas associated with unattended modalities (Mozolic et al., 2012).

Deficits in attentional control in the elderly population could then be assumed to take part in the increased amount of multisensory information being processed. OA fail to focus on one stimulus but rather integrate all the information available to them. However, several studies found that OA were still able to engage selective attention in simple tasks (Townsend et al., 2006; Hugenschmidt et al., 2009a,b; Guerreiro et al., 2012, 2014, 2015; Fiacconi et al., 2013). As a consequence, selective attention cannot solely explain the increased MSI in OA.

Nevertheless, deficits in selective attention could explain the fact that OA were more distracted by stimuli within the same modality or in another modality as the attended stimulus (Furman et al., 2003; Poliakoff et al., 2006a; Townsend et al., 2006; Diederich et al., 2008; Hugenschmidt et al., 2009a; Peiffer et al., 2009; Guerreiro and Van Gerven, 2011; Setti et al., 2011a; Guerreiro et al., 2012, 2013, 2014, 2015; DeLoss et al., 2013; McGovern et al., 2014).

#### Increased Noise at Baseline

None of the hypotheses described above are entirely able to explain the increased MSI in the elderly population (Mozolic et al., 2012; Freiherr et al., 2013). Mozolic et al. (2012) developed another hypothesis explaining the differences between OA and YA: increased noise at baseline. The authors argued that when OA engaged in selective attention, multisensory areas activity was reduced but remained higher than the YA, leading to sensory noise. When YA engaged in selective attention, the multisensory areas enhancements in their brain were suppressed to successfully ignore non-relevant information. The authors explained that because of this noise, OA were less able to ignore distractors but when information from the environment became relevant, they benefited from this higher baseline and showed larger MSI responses. This is beneficial when all information is reliable, and a disadvantage when part of the information should be ignored. This hypothesis could explain why OA maximized the use of MSI but are still impaired regarding their performance in the task or when the task is more difficult (e.g., cognitively demanding). This hypothesis fits best to the results described in the systematic review.

#### Additional Results Not Described by Other Reviews

Five different hypotheses have been put forward by other authors to explain the differences between younger and OA regarding MSI: a general cognitive slowing, the inverse effectiveness, a larger time window of integration, deficits in attentional control and the one that fits best the results, the increased noise at baseline. However, this systematic review pointed to an age-related change that has not been described in the previous systematic reviews: a deficit in the weighing of sensory information in the elderly population compared to YA.

### Weighing of Sensory Information

This part describes the differences in the weighing of sensory information between OA and YA, the normal Bayesian integration occurring in the brains of YA, brain areas involved in weighing sensory information in YA, and finally, the relationship between weighing sensory information and OA.

#### Differences in the Weighing of Sensory Information

In this systematic review, we found that OA were impaired at properly weighing sensory information from the environment compared to YA (Stelmach et al., 1989; Teasdale et al., 1991; Strupp et al., 1999; Redfern et al., 2001, 2009; Allison et al., 2006; Poliakoff et al., 2006a; Deshpande and Patla, 2007; Hugenschmidt et al., 2009a; Elliott et al., 2011; Guerreiro and Van Gerven, 2011; Berard et al., 2012; Dobreva et al., 2012; Wu et al., 2012; DeLoss et al., 2013; Guerreiro et al., 2013; Bisson et al., 2014; Deshpande and Zhang, 2014; Eikema et al., 2014; McGovern et al., 2014; Brodoehl et al., 2015). The experiments that revealed this finding encompassed tests in which the sensory information was disrupted or taken away or when distractors were included. These age-related changes have not been described in the reviews that we found on age-related effects on MSI (Mozolic et al., 2012; Freiherr et al., 2013), probably because the previous reviews mostly focused on audiovisual tasks with a static position (Mozolic et al., 2012; Freiherr et al., 2013) while this effect was particularly observable when wrong information was presented during postural tasks involving visual, somatosensory and vestibular information. For example, Bates and Wolbers (2014) showed that OA relied less than predicted on visual landmarks in a navigation task. This was also seen when a GVS was added to help the subjects reduce postural sway: OA were unable to properly use the added information (Eikema et al., 2014).

The changes in the weighing of the information could be caused by a failure in detecting that the information is important or unreliable, and/or in a failure in inhibiting the use of unreliable information. This would be consistent with Mozolic et al. (2012) hypothesis, the increased noise at baseline in the elderly population leads to sensory noise and could hinder them judging if the information is irrelevant or unreliable. OA were shown to recruit more multisensory brain areas than YA (Townsend et al., 2006; Heuninckx et al., 2008; Venkatraman et al., 2010), specifically frontal areas (Freiherr et al., 2013). These areas are known to be related to the selection of multisensory stimuli (Talsma, 2015) and connected to each other, leading to difficulties to downregulate individual modalities and irrelevant information (Berard et al., 2012; Mozolic et al., 2012). Besides, using all information available could be a good strategy for OA in whom one or more sensory sources have become unreliable due to bad unimodal processing, as this strategy could compensate for lower level sensory degradation (Berard et al., 2012).

#### Bayesian Integration

The brain process of weighing sensory information from the environment follows the principle of Bayesian integration (Ernst, 2006; Bates and Wolbers, 2014; Ursino et al., 2014). This process aims to increase the accuracy of the percept by reducing its uncertainty (Bates and Wolbers, 2014). Stimulus information comes to a person through different modalities, for instance, the size of an object can be estimated through vision and haptics. The Bayesian model assumes that the brain weighs each signal optimally with respect to its variance and combines them into one estimate with a smaller variance than the variance of the individual estimates (Ernst, 2006; Ursino et al., 2014). According to the maximum likelihood estimation, the reliability of the combined estimate is the sum of the individual estimates (Ernst, 2006), i.e., it is generally valuable to integrate stimuli from different modalities as OA seem to do.

#### Brain Areas Involved in Weighing Sensory Information

It has been shown that the human brain seems to integrate cue information in a Bayesian optimal manner. Computational studies have been done considering different areas of the brain (Anastasio et al., 2000; Colonius and Diederich, 2004b; Gu et al., 2008; Ursino et al., 2014). The authors found that the neurons present in the SC and the dorsal medial superior temporal area use the Bayesian rule to integrate and weigh multisensory information to arrive at a least variable percept.

#### Weighing Sensory Information and OA

The results described earlier suggest that OA are impaired at properly weighing sensory information from the environment. Additionally, Bates and Wolbers (2014) showed that OA relied less than optimally expected on visual information in a navigation task by using Bayesian modeling as described above. Age-related changes in the brain, for instance, gray or white matter losses (Peters, 2006; Hedman et al., 2012) or differences in brain activity (Cabeza, 2002; Peters, 2006; Greenwood, 2007; Park and Reuter-Lorenz, 2009) could have led to this degradation in the integration of the sensory cues present in the environment of OA. However, this level of integration could still be optimal for the performance of elderly people given their degradation of unisensory perception (Teasdale et al., 1991; Bugnariu and Fung, 2007; Owsley, 2011).

These findings will need further investigation with new experiments in order to better understand age-related changes in MSI in the healthy elderly population.

## Strengths and Limitations

This systematic review has been written following the guidelines of the PRISMA statement (Moher et al., 2009) that aims to improve the reporting of systematic reviews. The literature research has been done in Scopus, a database covering Medline, Embase and Compendex (Burnham, 2006) increasing the number of potentially relevant articles. Furthermore, the articles had to have sufficient quality according to the NOS (Wells et al., 2012) to be included in the analysis of the results. Finally, this review shows results that have not been reported before, to our knowledge, in other reviews.

However, some articles on MSI in the healthy elderly population might not be included in the search results. This is due to the search limits such as timeframe of the search. These articles are likely to concern more recent publication dates. Another reason might be that researchers did not consider their research as MSI research, and therefore did not label it as such. We know of at least two articles, which we have previously read, that were interesting for our aim, but did not come up in the search results (Mazaheri et al., 2015; Yeh et al., 2015), and were therefore not included in the systematic review.

Another potential limitation is that only one reviewer (AD) did the search of articles in Scopus and the selection of the full-text articles in the systematic review. A double checking by another reviewer (or reviewers) might help to avoid mistakes and bias in the screening and in the selection of the articles.

## Conclusion and Future Directions

The main finding of this systematic review is the fact that OA encounter difficulties in properly weighing information from different sensory modalities or in selective MSI and are more hindered by a second task. OA use all information available, even if they should not (e.g., distractors, disrupted information). These results were found for all combinations of modalities described in this review and in several tests, including the sound-induced flash illusion, n-back tasks with distractors, walking and navigation tasks, postural tasks with or without cognitive dual task, selective attention task with distractors, visual straight ahead tasks, control of movement timing tasks and visual-vestibular task with a cognitive dual task. In these tests, sensory information was disrupted or taken away, or distractors were included. The hypothesis of increased noise at baseline described by Mozolic et al. (2012) explaining the differences between OA and YA in MSI seems most accurate to explain why OA have trouble in properly weighing sensory information. Other explanations are plausible as well but cannot explain the full set of results or are too general to be of use in a clinical setting.

The results of the review suggests that accurately diagnosing MSI issues in the elderly population could be helpful to predict and understand problems in ADLs in the elderly population which could have an impact on their everyday life and on society. Since the tests reviewed here were applied in laboratory settings on small groups, they are not readily available or applicable for clinical practice. The large number of available tests (*n* = 69) identified in this review is a good starting point to develop a clinically useful tool or toolkit assessing MSI in the healthy elderly population with the aim to aid early diagnosis. In the future, this toolkit could help in early detection and to develop a more targeted intervention in clinical practice.

## Author Contributions

ALD developed the research strategy by selecting the database, proper keywords, limitations, and quality assessment tool used for the literature selection. She did the literature research, screened the articles found and selected or rejected them as well as the summary of the articles selected. She analyzed and summarized the results and wrote the review. PCS helped at each stage of the literature research and with the structure and writing of the article. She participated in the discussion of the results. A-MB and JBFE participated in the development of the research strategy, in the discussion of the results and reviewed the article.

## Funding

This research was performed in the context of the PACE (Perception and Action in Complex Environments) project, which is funded from the European Union’s Horizon 2020 research and innovation program under the Marie Sklodwska-Curie grant agreement No 642961.

## Conflict of Interest Statement

The authors declare that the research was conducted in the absence of any commercial or financial relationships that could be construed as a potential conflict of interest.

## Footnotes

^1^http://www.crd.york.ac.uk/PROSPERO/display_record.asp?ID=CRD42016036946

## Abbreviations

ADLs: Activities of daily living
Basic ADL: Basic activities of daily living
EEG: Electroencephalography
fMRI: Functional magnetic resonance imaging
GVS: Galvanic vestibular stimulation
IADL: Instrumental activities of daily living
LED: Light-emitting diode
MEG: Magnetoencephalography
MRI: Magnetic resonance imaging
MSI: Multisensory integration
OA: Older adults
SC: Superior colliculus
SOA: Stimulus onset asynchrony
SOT: Sensory organization test
STS: Superior temporal sulcus
YA: Younger adults.
