# Supplementary material for: Effects of Aging in Multisensory Integration: A Systematic Review
Source: Front Aging Neurosci. 2017 Mar 28;9:80. doi: 10.3389/fnagi.2017.00080 (PMC5368230; doi:10.3389/fnagi.2017.00080)
Supplement: Supplementary file 1 [file DataSheet_1.docx]

Appendix 1 (Uploaded as a separate file)

Table 3: Studies (n=53) investigating MSI in the healthy elderly population rated with the Newcastle-Ottawa Quality Assessment Scale (NOS)(Wells et al., 2012).

| **Study** | **Quality assessment criteria** | | | | | | | |  |
| --- | --- | --- | --- | --- | --- | --- | --- | --- | --- |
|  | ***Selection (4 stars)*** | | | | ***Comparability (2 stars)*** | | ***Exposure (2 stars)*** | | ***Quality score (Maximum = 8 stars)*** |
|  | Is the population definition adequate? | Representativeness of the population | Selection of controls | Definition of controls | Study controls for age | Study controls for additional factor (5) | Same method of ascertainment for cases and controls | Non-response rate |  |
|  | Healthy, without any known disease, 60+ years old | Representative of the healthy elderly population | Same community | No current severe or chronic disease | Older adults compared to younger adults | Gender, education, intelligence, level of cognition | Methods are identical | Same rate for both groups |  |
| (Allison et al., 2006) | * | * | * | * | * | - | - | * | 6 |
| (Bates and Wolbers, 2014) | * | * | - | * | * | * | * | * | 7 |
| (Bellomo et al., 2009) | * | * | * | * | - | * | * | * | 7 |
| (Berard et al., 2012) | * | - | * | * | * | - | * | * | 6 |
| (Bisson et al., 2014) | * | - | - | * | * | * | * | * | 6 |
| (de Boer-Schellekens and Vroomen, 2013) | * | - | * | * | * | - | * | * | 6 |
| (Brodoehl et al., 2015) | * | * | - | * | * | * | * | * | 7 |
| (Campbell et al., 2010) | - | - | * | - | * | * | * | * | 5 |
| (Cham et al., 2007) | * | * | - | * | - | * | * | * | 6 |
| (Chan et al., 2014a) | - | * | * | - | * | * | * | * | 6 |
| (Coté and Schaefer, 1981) | - | * | * | - | * | * | * | * | 6 |
| (Cui et al., 2010) | * | * | - | * | * | * | * | * | 7 |
| (DeLoss et al., 2013) | * | * | * | * | * | * | * | * | 8 |
| (Deshpande and Patla, 2007) | * | * | * | * | * | * | * | * | 8 |
| (Deshpande and Zhang, 2014) | * | * | - | * | * | * | * | * | 7 |
| (Diederich et al., 2008) | - | * | * | - | * | * | - | * | 5 |
| (Dobreva et al., 2012) | * | * | * | * | * | * | * | * | 8 |
| (Eikema et al., 2014) | * | * | - | * | * | * | * | * | 7 |
| (Elliott et al., 2011) | * | * | * | * | * | * | * | * | 8 |
| (Fiacconi et al., 2013) | * | - | - | * | * | - | * | * | 5 |
| (Furman et al., 2003) | * | * | - | * | * | * | * | * | 7 |
| (Guerreiro and Van Gerven, 2011) | * | - | * | * | * | * | * | * | 7 |
| (Guerreiro et al., 2012) | - | * | * | - | * | * | * | * | 6 |
| (Guerreiro et al., 2013) | - | * | * | - | * | * | * | * | 6 |
| (Guerreiro et al., 2014) | * | * | * | * | * | * | * | * | 8 |
| (Guerreiro et al., 2015) | - | * | * | - | * | * | * | * | 6 |
| (Hugenschmidt et al., 2009a) | * | * | - | * | * | * | * | * | 7 |
| (Hugenschmidt et al., 2009b) | * | * | - | * | * | * | * | * | 7 |
| (Hugenschmidt et al., 2014) | * | * | - | * | * | * | * | * | 7 |
| (Mahboobin et al., 2007) | * | * | - | * | * | * | * | * | 7 |
| (Mahoney et al., 2012) | * | - | - | * | * | * | * | * | 6 |
| (Mahoney et al., 2011) | * | * | - | * | * | * | * | * | 7 |
| (McGovern et al., 2014) | * | * | * | - | * | * | * | * | 7 |
| (Norman et al., 2006) | * | * | - | * | * | * | - | * | 6 |
| (Oscar-Berman et al., 1990) | * | - | * | * | * | * | * | * | 7 |
| (Peiffer et al., 2007) | * | * | - | * | * | * | * | * | 7 |
| (Poliakoff et al., 2006a) | * | - | * | * | * | - | * | * | 6 |
| (Poliakoff et al., 2006b) | * | * | - | * | * | * | * | * | 7 |
| (Redfern et al., 2001) | * | * | - | * | * | * | * | * | 7 |
| (Redfern et al., 2009) | * | * | - | * | * | * | * | * | 7 |
| (Setti et al., 2011a) | * | * | * | * | * | * | * | * | 8 |
| (Setti et al., 2011b) | * | * | * | * | * | * | * | * | 8 |
| (Stelmach et al., 1989) | * | * | - | * | * | * | * | * | 7 |
| (Stephen et al., 2010) | - | * | - | - | * | * | * | * | 5 |
| (Strupp et al., 1999) | * | * | - | * | - | * | * | * | 6 |
| (Teasdale et al., 1991) | * | * | * | * | * | * | * | * | 8 |
| (Temprado et al., 2013) | * | * | * | * | * | * | * | * | 8 |
| (Townsend et al., 2006) | * | * | * | * | * | * | * | * | 8 |
| (Wu et al., 2012) | * | - | - | * | * | * | * | * | 6 |
| Articles excluded from the summary of the results (Scores <5) | | | | | | | | | |
| (Chan et al., 2014b) | - | - | * | - | * | - | * | * | 4 |
| (Cohen et al., 2014) | * | - | - | * | - | - | * | * | 4 |
| (Prioli et al., 2005) | - | - | - | - | * | - | * | * | 3 |
| (Woollacott et al., 1987) | - | - | - | - | * | - | * | * | 3 |

(*) = Acceptable; (-) = Not acceptable

The studies were included in the summary if their sum score on the NOS was equal or superior to five stars out of eight. The studies that failed to reach five stars were excluded from the summary of the results.

Appendix 2 (Uploaded as a separate file)

Table 4: Summary of tests and results of the articles included in the systematic review (n=53).

| ***Tests on the visual and auditory modalities*** | | | | | | | | | | |  |
| --- | --- | --- | --- | --- | --- | --- | --- | --- | --- | --- | --- |
| **Title, first author, year of publication** | **Study characteristics** | | | | | | | | | **Results related to the effects of aging** |  |
|  | **Participants (number; age range; mean(SD); percentage of men)** | | | **Material** | **Experiments** | | | | |  |  |
|  | **Older adults** | **Young adults** | **Other groups** |  | **Tasks** | **Conditions** | **Instructions** | | **Time and repetition** |  |  |
| Multisensory integration compensates loss of sensitivity of visual temporal order in the elderly  de Boer-Schellekens, 2013 | 11; 60–69; 65.4(3.6); n.r. | 10; 20–29; 22.1(3.2); n.r. | 9; 30–39; 33(2.1); n.r.  10; 40–49; 44.1(2.6); n.r.  10; 50–59; 52.5(1.8); n.r. | Serial response box, headphones, computer with E-Prime, screen | Temporal order judgement | A) Visual  1) With white noise clicks  2) Without white noise clicks  B) Auditory  C) Audiovisual  Different SOA | Report the temporal order of the stimuli | | A) 576 trials  B) 160 trials  C) 160 trials | "Results showed that sensitivity of visual, auditory, and audiovisual temporal order declined from 50 years on. However, there was no corresponding decline in MSI as the click sounds actually compensated the loss of sensitivity of visual temporal order in the elderly. Sensitivity of audiovisual temporal order did not correlate with MSI, suggesting that well-preserved explicit judgments about cross-modal temporal order are not required for MSI to occur." |  |
| The effects of multisensory targets on saccadic trajectory deviations: Eliminating age differences  Campbell, 2010 | 14; 61–73; 67.1(4.2); n.r. | 14; 18–29; 20.9( 2.9); n.r. | - | Camera-based eye tracker, 2 speakers, chin rest, computer, screen | Sound localization tasks | A) Sound localization test  B) Sound localization with distractors, target appears on the left or right  1) With distractors below or above  a) Congruent  b) Incongruent  2) Without distractors | A) Judge whether the sound came from the left or from the right B) Move their gaze to the target with a single saccade | | 336 trials | “The results show that bimodal targets produced larger deviations away than unimodal targets, but only when the distractor preceded the target, and this effect was comparable across age groups. Furthermore, in contrast to previous research, older adults in this study showed similar deviations away from distractors to those of younger adults.” |  |
| Older age results in difficulties separating auditory and visual signals in time.  Chan, 2014a | 13; 61–72; 66(n.r.); 53.9% | 15; 21–32; 25(n.r.); 33.3% | - | Response box, headphones, chin rest, r, computer with ViSaGe, cathode ray tube monitor | Audio-visual asynchrony judgment with sound-lead or sound-lag asynchrony | A) Visual  B) Auditory  C) Audiovisual  Different SOA | Discriminate between two intervals which one contains the asynchronous stimulus | | 12 trials | A main effect of aging was found for the detection threshold (p< 0.001). "The older adults have a larger mean sound-lead asynchrony detection threshold and a larger mean sound-lag threshold only for low frequency sound stimulus at the suprathreshold stimulus level compared to young adults." |  |
| Advancing age alters the influence of eye position on sound localization  Cui, 2010 | 10; 66–81; 73.9(5.6); 40% | 11; 18–39; 26.7(6.5); 45.5% | 9; 40–65; 53.8(5.6); 44.4% | Fully enclosed, echo- attenuated room, 3-thick SonexTextile acoustic panels, servo-controlled robotic arm, joystick, laser-LEDs, miniature red LED, loudspeaker, key press, bite-bar, computer, cylindrical screen | Localization tasks | A) Control condition  B) Eccentric condition | A) Align the beam of a laser pointer with the perceived sound location and signal their localization with a key press  B) Use peripheral vision to locate the target | | 15-20min of fixation, 693 trials | “All three age groups demonstrated a time-dependent shift of auditory space in the direction of eye position. Moreover, this adaptation showed a clear decline with advancing age, but only for peripheral auditory space (beyond ±10° from midline).” |  |
| Multisensory integration, aging, and the sound-induced flash illusion  DeLoss, 2013 | Exp 1:  12; 75.7(5.1); 50%  Exp 2:  24; 72.5(4.3); 62.5% | Exp 1:  12; 20.8(0.6); 58.3%  Exp 2:  24; 22(0.8);  45.8% | - | Chin rest, keyboard, computer, screen | Sound-induced flash illusion and go/no go tasks | A) Experiment 1  1) Pre-test  2) Experiment  a) Visual block 1-3 flashes  b) Auditory block 1-3 beeps  c) Audiovisual block 1-3 flashes paired with 0-3 beeps  B) Experiment 2  1) Visual go/no-go cue  2) Auditory go/no-go cue  3) No cue | A) 1) Discriminate flashes and beeps  2) Report the perceived number of flashes or beeps  B) No go if the frequency of the tone is lower or if the size of the disc increased | | 3h | "Older participants demonstrated greater multisensory integration, a greater influence of the beeps when judging the number of visual flashes than younger observers. In the visual go/no-go task they found a decrease in the illusion, yet in the auditory go/no-go task they found an increase in the illusion. These results demonstrate that older individuals exhibit increased multisensory integration compared with younger individuals." |  |
| Assessing age-related multisensory enhancement with the time-window-of-integration model Diederich, 2008 | 15; 65–75; 69.6(n.r.); 40% | 6; 20–22; n.r.(n.r.); 50% | - | Completely darkened and sound attenuated room, 2 Speakers, fixation LED, 2 LEDs on the loudspeakers, chin rest | Selective attention tasks | A) Visual  B) Auditory  C) Audiovisual  Different SOA | Gaze to the target as quickly as possible while ignoring any auditory non-targets that could happen before or after the stimulus onset | | 2.5h for young adults, 4h for older adults, 960 trials | “The response time pattern for both groups was similar. The elderly participants were considerably slower than the younger participants under all conditions but showed a greater multisensory enhancement, that is, they seem to benefit more from bimodal stimulus presentation.” |  |
| Influence of age, spatial memory, and ocular fixation on localization of auditory, visual, and bimodal targets by human subjects  Dobreva, 2012 | 15; 65–81; n.r.(n.r.); 46.7% | 12; 18–30; n.r.(n.r.); 50% | - | Electrooculography, darkened room lined with echo-attenuating foam, robotic arm, visually guided manual laser pointing with two- axis joystick, two laser light-emitting diodes (LED) (red and green), rigidly mounted 60 cm above the subject’s head, two-way coaxial loudspeaker with red LED, personalized bite-bar, keyboard | Localization of auditory, visual and bimodal targets | A) Ongoing targets with target fixation  1) Visual  2) Auditory  B) Central fixation  1) Ongoing  2) Transient  C) Transient target with target fixation  1) Visual  2) Auditory  D) Transient bimodal targets with target fixation | A) Align laser pointer with perceived target location  B) Localization with peripheral vision  C) Guided pointer with vision to the position remembered  D) Report the location of the perceived auditory component | | 2 sessions, 376 trials | “In comparison with young adults, elderly subjects showed (1) worse precision in most paradigms, especially when localizing with peripheral vision under central fixation; (2) greatly impaired vertical localization of auditory and bimodal targets; (3) increased horizontal overshoot in the central field for remembered visual and bimodal targets across response delays; (4) greater vulnerability to visual bias with bimodal stimuli.” |  |
| The influence of aging on audiovisual temporal order judgments.  Fiacconi, 2013 | Exp 1:  21; 70-82; 73.9(n.r.); n.r.  Exp 2:  12; 70-82; 75.2(n.r.); n.r. | Exp 1:  23; 18-29; 22.1(n.r.); n.r.  Exp 2:  12; 21-31; 25.5(n.r.); n.r. | - | 2 speakers, chin\forehead rest, computer, screen | Audiovisual temporal order judgment | A) Visual and auditory stimuli presented from the same perceived location  B) Presented from different locations | Judge which stimulus was presented first | | 45min | “The authors found no effect of stimulus location, and no evidence of age-related declines in performance in either experiment.” |  |
| Now You See It, Now You Don’t: Evidence for Age-Dependent and Age-Independent Cross-Modal Distraction  Guerreiro, 2011 | 30; 61–77; 67.7(5.5); 66.7% | 30; 20–29; 22.3(2.2); 10% | - | Eye-tracker, headphones, response box, computer, screen | N-back tasks | A) Visual n-back task (n= 0 to 2)  1) With auditory distraction  2) Without auditory distraction  B) Auditory n-back task (n=0 to 2)  1) With visual distraction  2) Without visual distraction | Judge whether the current digit or spoken number was the same as n positions back in the sequence | | 480 trials | “Irrelevant auditory information did not disrupt accuracy in the visual n-back task in either age group, Visual distraction disrupted accuracy on the auditory n-back task, especially in older adults.” |  |
| Automatic selective attention as a function of sensory modality in aging  Guerreiro, 2012 | 25; 60–76; 68.8(4.9); 40% | 30; 20–26; 22.4(1.9); 16.7% | - | Eye-tracker, keyboard, computer, screen | Localization of auditory, visual and bimodal targets | A) Visual cue and visual target  B) Auditory cue and auditory target  C) Visual cue and auditory target  D) Auditory cue and visual target | Modality matching task: Respond to each stimulus as fast as possible  Spatial cueing task: Respond to red and low pitched tone or to green and high pitched tone | | 448 trials | “The results showed facilitation (shorter reaction times with valid relative to invalid cues at shorter SOAs) in the unimodal auditory and in both cross-modal tasks but not in the unimodal visual task. In contrast, there was IOR (longer reaction times with valid relative to invalid cues at longer SOAs) in both unimodal tasks but not in either of the cross-modal tasks. Most important, these spatial cueing effects were independent of age.” |  |
| Making sense of age-related distractibility: The critical role of sensory modality Guerreiro, 2013 | 22; 60–73; 65.4(3.7); 45.5% | 24; 20–27; 21.7(2.3); 33.3% | - | Sound-attenuated chamber, speaker, keyboard, computer, screen | Audiovisual n-back tasks | A) Visual n-back task (n=1 or 2)  1) With auditory distraction  2) With visual distraction  3) Without distraction  B) Auditory n-back task (n=1 or 2)  1) With auditory distraction  2) With visual distraction  3) Without distraction | Judge whether every newly presented digit was the same or not as n position back | | 2 Sessions, 480 trials | “Whereas reaction time data indicated that both young and older adults are particularly affected by unimodal distraction, accuracy data revealed that older adults, but not younger adults, are vulnerable to cross-modal visual distraction. These results support the notion that age-related distractibility is modality dependent.” |  |
| Age-equivalent top–down modulation during cross-modal selective attention Guerreiro, 2014 | 20; 62–80; 68.7(5.1); 50% | 20; 19–29; 24.1(3); 50% | - | Electroencephalography (EEG), press buttons, speakers, computer, screen | Selective attention task with EEG and a post-experiment recognition task | A) Remember faces and ignore voices  B) Remember voices and ignore faces  C) Passively view and hear | A) and B) Press different button if the probe matched a cue stimuli or not.  C) Indicate the direction of an arrow | | 60 trials | “We found top-down modulation of visual processing was observed as a trend toward enhancement of visual information in the setting of auditory distraction, but no significant suppression of visual distraction when auditory information was relevant. Top-down modulation of auditory processing, on the other hand, was observed as suppression of auditory distraction when visual stimuli were relevant, but no significant enhancement of auditory information in the setting of visual distraction. In addition, greater visual enhancement was associated with better recognition of relevant visual information, and greater auditory distractor suppression was associated with a better ability to ignore auditory distraction. There were no age differences in these effects, suggesting that when relevant and irrelevant information are presented through different sensory modalities, selective attention remains intact in older age.” |  |
| Top-down modulation of visual and auditory cortical processing in aging  Guerreiro, 2015 | 16; 60–71; 65.3(3.9); 31.3% | 16; 20–29; 23.3( 3); 43.8% | - | Functional magnetic resonance imaging (fMRI), keyboard, speakers, computer, screen | Selective attention task | A) Localizer run  B) Experimental runs  1) Perceptual baseline  2) Remember scenes  3) Remember faces  4) Remember voices  5) Remember music  C) fMRI task: unexpected memory task of the stimuli | A) Passively hear and view stimuli  B) 1) Indicate the direction of an arrow  2) to 5) Indicate if probe stimulus matched one of the previously presented cue stimuli  C) Remember stimuli | | A) 1,2min  B) 50min  C) 480 trials | "We found no top-down modulation of auditory sensory cortical processing in either age group. In contrast, we found top-down modulation of visual cortical processing in both age groups, and this effect did not differ between age groups." |  |
| Suppression of multisensory integration by modality-specific attention in aging Hugenschmidt, 2009a | 20; n.r.; 73.3(n.r.); 45% | 21; n.r.; 26.6(n.r.); 57.1% | - | Sound and light attenuated booth, response buttons, chin rest, computer, screen | Spatial localization task | A) Endogenous: attentional cue prior to the target  1) Visual cue-visual target  2)Visual-audiovisual  3) Auditory-auditory  4) Auditory-audiovisual  5) Audiovisual-auditory  6) Audiovisual-visual  7) Audiovisual-audiovisual  B) Exogenous: No cue  1) Visual  2) Auditory  3) Audiovisual | Press a button if they see or hear a target in one of the two location | | 2 sessions, 240 trials | “Older adults had greater multisensory integration than younger adults in all conditions, yet were still able to reduce integration using selective attention. This suggests that attentional processes are intact in older adults, but are unable to compensate for an overall increase in the amount of sensory processing during divided attention.” |  |
| Preservation of crossmodal selective attention in healthy aging  Hugenschmidt, 2009b | 26; n.r.; 67.9(3.5); 42.3% | 26; n.r.; 28.3(5.9); 50% | - | Sound and light attenuated booth, array of four speakers and four red LEDs, 5 LEDs, speakers, chin rest, computer, screen | Selective attention tasks | Visual attentional cue:  1) Two eyes  2) Two ears  3) One eye, one ear  Target:  1) Visual  2) Auditory  3) Audiovisual | Make a choice between the color red and blue | | 444 trials | “All analyses showed that older adults benefited behaviorally from selective attention in both visual and auditory conditions, including robust suppressive effects of attention. Of note, the performance of the older adults was commensurate with that of younger adults in almost all analyses, suggesting that older adults can successfully engage crossmodal attention processes. Thus, age-related increases in distractibility across sensory modalities are likely due to mechanisms other than deficits in attentional processing.” |  |
| The sound-induced flash illusion reveals dissociable age-related effects in multisensory integration  McGovern, 2014 | 25; 65–88; 71(n.r.); 36% | 25; 18–30; 24(n.r.); 40% | - | Darkened, windowless room, key press, headphones, chin rest, computer, screen | Sound-induced flash illusion | One or two flashes accompanied by one, two or no auditory beeps with different stimulus onset asynchrony (SOA) | Report the number of flashes | | 25-30min | "The elderly are significantly more variable in their susceptibility to the fission illusion" (single visual flash accompanied by two auditory tones is perceived as two flashes) than young. "No equivalent age-related changes in susceptibility to the sound-induced fusion illusion" (two visual flashes accompanied by a single auditory tone are perceived as one flash). |  |
| Age-related multisensory enhancement in a simple audiovisual detection task  Peiffer, 2007 | 23; 65-80; n.r.(n.r.); 52.2% | 27; 18-38; n.r.(n.r.); 48.1% | - | Sound and light attenuated booth, 4 speakers, 2 LEDs | Audiovisual detection task | A) Visual  B) Auditory  C) Audiovisual | Press a key if hear or see something | | 120 trials | “No significant differences in unisensory response times were seen; however, older adults actually showed faster multisensory responses than younger adults.” |  |
| Is inefficient multisensory processing associated with falls in older people?  Setti, 2011a | 16; n.r.; 60 and older; 56.3% | 16; n.r.; 24.4(4);  43.8% | Fall-prone older adults: 16; n.r.; 60 and older; 25% | Loudspeakers, response key, chin rest, computer, screen | Sound-induced flash illusion | A) Visual: 1 or 2 flashes  B) Auditory 2 beeps  C) Audiovisual  1) Congruent: 1 flash/1 beep or 2 flashes/2 beeps  2) Illusory: Onset of beep precede or follow flash  Different SOA | Report the number of flashes, if no flashes, report the number of beeps | | 162 trials | “Healthy older adults did not show as much of a decrease in the frequency at which the illusion was experienced as the young participants from 70 to 270ms, although the two groups overall did not statistically differ in the amount of illusion experienced.” |  |
| Audiovisual temporal discrimination is less efficient with aging: An event-related potential study  Setti, 2011b | 18; n.r.; 71(5.4); 61.1% | 18; n.r.; 24(3.2); 33.3% | - | EEG, headphones, computer, screen | Audiovisual temporal discrimination and an EEG examination | Audiovisual, different SOA | Report which stimulus was presented first | | 176 trials | “No effect of age on the temporal order judgment accuracy at a SOA of 70ms whereas, at 270ms SOA, older adults were less accurate than younger (same for the amplitudes of EEG)”. |  |
| Aging-related changes in auditory and visual integration measured with MEG  Stephen, 2010 | 8; 65-78; n.r.(n.r.); 37.5% | 8;20-33; n.r.(n.r.); 50% | - | Ear tubes using etymotic transducers, Electrooculography, Magnetoencephalography(MEG), Magnetic resonance imaging (MRI), finger button press, computer, screen | Near, far localization task | A) Visual, soccer ball presented far or close  B) Auditory, near or far manipulations  C) Audiovisual, with the two modalities never presented in a conflicting manner | Respond with left or right button when stimuli were near or far | | 720 trials | “The mean reaction times of the elderly were significantly slower than the young. In addition, in the young we found significant facilitation of reaction times to the multisensory stimuli relative to both unisensory stimuli, when comparing the cumulative distribution functions, which was not evident for the elderly. The elderly had larger amplitude responses (∼100ms) to auditory stimuli relative to the young when auditory stimuli alone were presented, whereas the amplitude of responses to the multisensory stimuli was reduced in the elderly, relative to the young.” |  |
| Changing channels: An fMRI study of aging and cross-modal attention shifts  Townsend, 2006 | 10; 65-89; 70.7(7); 40% | 10; 18-41; 27.9(8); 40% | - | fMRI, MRI-compatible headphones, custom made response device, computer, screen | Auditory-visual attention task with fMRI | A) Focus visual: light blue target  B) Focus auditory: high tone target  C) Bimodal: bimodal shift cues “HEAR” or “LOOK” and focus on visual or auditory | A) and B) Press button when the target appears  C) Identify the target stimulus with the cue, press button when the target appears | | 336 trials | “Older adults performed as well as the younger adults, but showed age-related differences in BOLD responses. The most striking of these differences were bilateral frontal and parietal regions of significantly increased activation in older adults during both focused and shifting attention.” |  |
| Age-related multisensory integration elicited by peripherally presented audiovisual stimuli  Wu, 2012 | 15; 60-78; 68.6(n.r.) n.r. | 15; 22-28; 23.9(n.r.); n.r. | - | Dimly lit, sound-attenuated and electrically shielded room, earphone, keyboard, computer, screen | Localization of auditory, visual and bimodal targets | A) Visual  B) Auditory  C) Audiovisual | Indicate if the target appeared in the left or in the right hemispace | | 25min, 4500 trials | “The time window of audiovisual behavioral facilitation in the elderly participants was longer but more delayed than that of the younger participants.” |  |
|  | | | | | | | | | | |  |
| ***Tests on the visual, vestibular and somatosensory modalities*** | | | | | | | | | | |  |
| **Title, first author, year of publication** | **Study characteristics** | | | | | | | | | **Results of aging** |  |
|  | **Participants (number; range; mean(SD); percentage of men)** | | | **Material** | **Experiments** | | | | |  |  |
|  | **Older adults** | **Younger adults** | **Other groups** |  | **Tasks** | **Conditions** | | **Instructions** | **Time and repetition** |  |  |
| Multisensory reweighting of vision and touch is intact in healthy and fall-prone older adults  Allison, 2006 | 15; n.r.; 79(3); n.r. | 10; 19-28; n.r.(n.r.); n.r. | Fall-prone older adults: 28; n.r.; 83(4); n.r. | Multisensory moving room, force plate, googles that limit vision, smooth circular metal plate, tripod, computer controlled servomotor, strain gauges, tracking sensors with headband, ultrasound position tracking system, snug harness, computer, screen | Postural task | Moving visual stimulus projected onto a screen in a moving room. Participants held their fingertip touching a moving touch plate. Touch amplitude (mm): visual amplitude (mm)  A) 8:2  B) 4:2  C) 2:2  D) 2:4  E) 2:8 | | Maintain posture | 7h | "No group differences in overall levels of vision and touch gain were found. Both healthy and fall-prone older adults demonstrated the same pattern of adaptive gain change as healthy young adults. Like the young adults, both elderly groups displayed clear evidence of intra- and inter-sensory reweighting to both vision and touch motion stimuli. These data suggest that, for small amplitude vision and touch stimuli, the central sensory reweighting adaptation process remains intact in healthy and fall-prone older adults with sufficiently intact peripheral sensation." |  |
| How cognitive aging affects multisensory integration of navigational cues  Bates, 2014 | 22; 60-74; n.r.(n.r.); 50% | 24; 19-23; n.r.(n.r.); 50% | - | Blacked out room with illuminated LED lights arrays providing landmarks, walking frame, dark glasses, ear defenders | Navigation task | A) Neuropsychology assessments: National adult reading test for crystallized intelligence, Corsi block test, berg balance scale and Timed Up and Go test  B) Navigation task: Follow the targets  At target 3:  1) Stand  2) Disoriented by being turned quickly in a rotating chair | | B) Go from the start location to the target 1, then target 2, then target 3. While here, the landmarks switched off for 10s. Return to target 1 | 40 trials | "Findings revealed performance impairments in the older adults, suggestive of a higher noise in the underlying spatial representations. In addition, even though both groups integrated visual and self-motion information to become more accurate and precise, older adults did not place as much influence on visual information as would have been optimal." |  |
| Balance and posture in the elderly: an analysis of a sensorimotor rehabilitation protocol.  Bellomo, 2009 | 20; 65-75; n.r.(n.r.); 50% | 20; 55-65; n.r.(n.r.); 50% | - | Motorized platform, handles with sensors, lower back machine, treadmill, speakers, computer, screen | Rehabilitation training | A) Sensorimotor training (GrHu)  B) Training with rehabilitation protocol (GrCl)  After training, participants are evaluated for:  1) Walk at normal speed  2) Displacement of the center of balance  a) Eyes open  b) Eyes closed  3) Extension/flexion of the trunk  4) Energy cost, oxygen volume and heart rate on treadmill | | 1) Walk at normal speed  2) Maintain posture  3) flexion /extension of the trunk  4) Walk on the treadmill | 36 rehabilitation sessions, 3 months | "With regard to walking, there was an improvement in step symmetry for participants in the GrHu group compared to baseline (0.93+/-0.09 vs. 0.84+/-0.1; p<0.05). Further, all subjects in the GrHu group showed a significant reduction in the energy used during a 4-min walk. Analysis of stabilometry data also showed a significant improvement in balance for those in the GrHu group, which was independent of age or gender. The multisensory training approach yields an improvement of balance in the elderly, which reduces the risk of falls. The observed improvement is significantly greater than that seen with the classical training program." |  |
| Impact of aging on visual reweighting during locomotion Berard, 2012 | 10; n.r.; 76.2(3.1); n.r. | 10; n.r.; 23.5(4.7); n.r. | - | 12 x 8m walking area, helmet-mounted display unit, 39 reflective markers, 3 head markers, 12 cameras VICON, computer with CAREN-3 | Walking task | Subjects walked at normal or fast gait speed while viewing stereoscopically a 3D scene shown in a helmet-mounted display unit  A) No perturbation  B) No visual input  C) Visual perturbation: Focus of expansion of the scene gradually rotate to the right or left | | Walk straight | 18 trials | "The results show that healthy young adults are able to reweight or down-regulate visual information, but the older adults showed deviation in their walking trajectory and reorientations in body segments despite attempting to ignore the visual information presented. They only found speed effects for the young subjects, who improve their performance when walking fast." |  |
| Striatal dopamine denervation and sensory integration for balance in middle-aged and older adults  Cham, 2007 | 35; 41- 83; 65(13); 51.4% | | | Positron emission tomography, MRI, Equitest posture platform | Postural task and MRI | A) Sensory Organization Test (SOT)  1) Eyes open or closed  2) Environment fixed or moved  3) Floor fixed or moved  B) MRI | | A) Maintain posture | 15min for SOT | No young vs older SOT results. "The SOT Condition #3 findings suggest that, in normal aging, the central ability to inhibit balance destabilizing vision-related postural control processes depends at least partially on striatal dopaminergic pathways. In contrast, striatal dopaminergic denervation does not appear to impair the ability to disengage destabilizing proprioceptive inputs and to trigger the vestibular control system during challenging sensory perturbations (SOT Conditions #4-6)." |  |
| Visual-vestibular interaction during goal directed locomotion: Effects of aging and blurring vision  Deshpande, 2007 | 9; 65–85; n.r.(n.r.); 66.7% | 9; 20–35; n.r.(n.r.); 55.6% | - | Percutaneous bipolar GVS, electrodes, custom made blurring googles, visual target, 10 infrared emitting diodes, optotrack camera banks | Walking task | Participants walked straight to the target to the bit of a metronome  A) Vision conditions  1) Normal vision  2) Blurring vision  B) GVS conditions  1) No stimulation  2) 2t anode on the left side  3) 4t anode on the left side  4) 2t anode on the right side  5) 4t anode on the right side | | Walk straight following the auditory signal | 30 trials | "Older participants demonstrated increased coupling of the head and trunk segments irrespective of visual and vestibular perturbations. The results suggest that when visual information was available, the vestibular input reweighting was less effective in older individuals, as shown by the scaled responses to the GVS intensities and the inability to converge efficiently towards the target." |  |
| Trunk, head, and step characteristics during normal and narrow-based walking under deteriorated sensory conditions  Deshpande, 2014 | 15; 65–85; n.r.(n.r.); 46.7% | 15; 20–30; n.r.(n.r.); 53.3% | - | 2 optotrack camera banks, compliant working surface, bipolar galvanic vestibular stimulation (GVS), tap, 10 infrared emitting diodes | Walking task | Walking 6 meters straight ahead at normal speed  A) Path manipulations  1) Unconstrained  2) Narrow base: tape placed 25cm apart  B) Vestibular manipulations  1) With GVS  2) Without GVS  C) Somatosensory manipulation  1) Compliant walking surface  2) Firm walking surface | | Walk straight ahead at normal speed | 256 trials | “Aging reduced gait speed. Only visual information, even though normal, is not sufficient in older persons for precise head control in the Medio-lateral direction. Gait speed decreased in the narrow-based walking condition, with larger decrease in the elderly (by 6%). In the elderly head roll increased with perturbed vestibular information in impaired somatosensory condition (by 40.70%). In both age groups trunk roll increased under impaired somatosensation in the narrow-based walking condition (by 43.62%) but not in normal walking condition. Older participants adopted a more cautious strategy characterized by lower walking speed when walking on a narrow base and exhibited deteriorated integrative ability of the central nervous system for head control. Accurate lower limb somatosensation may play a critical role in narrow-based walking.” |  |
| Application of intermittent galvanic vestibular stimulation reveals age-related constraints in the multisensory reweighting of posture  Eikema, 2014 | 11; n.r.; 74.8(6.4); 54.5% | 12; n.r.; 24.9(6.4); 41.7% | - | Force platform, electromagnetic tracker, pair of cylindrical mechanical vibrators, Stimex electrodes, conduction gel-coated, active shutter googles, data-acquisition board, model 2200 constant current isolator, stereoscopic projection screen | Postural task | A visual stimulus that mimics optic flow is shown on a screen.  A) Quiet stance  B) GVS perturbation  For A) and B)  1) Pre-perturbation phase  2) Perturbation phase: oscillation of visual surround and bilateral Achilles tendon vibration  3) Post-perturbation phase  4) Visual perturbation only  5) Somatosensory perturbation only | | Maintain posture | 10min | “Results show intermittent GVS decreased the excessive postural sway induced by the concurrent visual and proprioceptive perturbation in young but not in elderly participants. It is suggested that GVS increases sensory reliance on the vestibular system while elderly adults are less able to exploit this stimulation in order to reduce the destabilizing effect of the multisensory perturbation on their posture.” |  |
| Graph theory analysis of functional brain networks and mobility disability in older adults  Hugenschmidt, 2014 | 48; n.r.; 72(5.1); 43.8% | 24; n.r.; 26.4(5.1); 37.5 | - | 1.5T echo speed horizon LX General Electric scanner with twin speed gradients and a neurovascular head coil, Timer, chair | Lower extremity functioning with MRI afterwards | Short Physical Performance Battery:  A) Balance tests  1) Side by side stand  2) Semi-tandem stand  3) Tandem stand  B) Gait speed test  C) Chair stand tests  1) Single chair stand  2) Repeated chair stand | | A) Maintain posture  B) Walk to the other end of the course  C) Stand and sit on the chair | 30min | "Older adults with poorer mobility function exhibited reduced consistency of somatomotor community structure and a greater number of secondary connections with vestibular and multisensory regions of the brain." |  |
| Attention influences sensory integration for postural control in older adults  Redfern, 2001 | 18; 70–85; 74(3.2); 44.4% | 18; n.r.; 22.8(1.8); 55.6% | - | Posture platform Equitest, force sensors in the floor, hand-held microswitch, green LED, headphones | Postural task | A) Postural conditions  1) Seated  2) Fixed floor and stable visual scene  3) Sway-referenced floor and stable visual scene  4) Fixed floor and sway-referenced visual scene  5) Sway-referenced floor and sway-referenced visual scene  B) Information processing task  1) No information processing  2) Visual-simple reaction time  3) Auditory simple reaction time  4) Inhibition reaction time | | Maintain posture | 8h | "Performing a reaction time task was associated with increased postural sway in older subjects, but not in young subjects. The greatest influence of reaction time task on sway of older subjects was found during the sway-referenced floor/sway-referenced scene condition. Conversely, postural condition had an influence on reaction time task performance in both young and older subjects. The information processing task was increased in both young and older subjects only during the sway-referenced floor/scene condition." |  |
| Perceptual inhibition is associated with sensory integration in standing postural control among Older adults  Redfern, 2009 | 24; 70 – 82; 74.2(4.4); 50% | 24; 21 – 34; 25.7(3.8); 45.8% | - | Dynamic posturography platform, force sensors, key press, computer, screen | Postural task | A) Postural task  1) Visual conditions  a) Eyes open in the light  b) Eyes open in the dark  c) Sway-referenced visual scene  2) Platform conditions  a) Fixed support surface  b) Sway-referenced floor  B) Inhibition task  1) Perceptual task  a) Congruent side arrow pointed and position arrow  b) Incongruent  2) Motor task | | A) Maintain posture  B) 1)Press a button on the side an arrow pointed  2) Press the button on the side toward the arrow pointed or on the side opposite the arrow pointed | 54min, 18 trials | "In the older adults, perceptual inhibition was positively correlated with sway amplitude on a sway-referenced floor and with a fixed visual scene (r = .68, p < .001). Motor inhibition was not correlated with sway on either group. Perceptual inhibition may be a component of the sensory integration process important for maintaining balance in older adults." |  |
| Age related decline in postural control mechanisms  Stelmach, 1989 | 8; 60-76; 67(n.r.); 50% | 8; 21-26; 23(n.r.); 50% | - | Perturbation platform with hydraulic servo-mechanism, angular precision potentiometer, force transducers, pre-spaced Ag-AgCl electrodes, oscilloscope, hand-holder response key | Postural task | A) Platform rotations  1) Large/ fast  2) Small/slow  B) Eyes conditions  1) Eyes closed  2) Eyes open | | Maintain posture. Press a button as soon as they detect platform movements | 30 trials | "Elderly take longer to detect postural disturbance (p<0,01) and would be further from their basis of support when reacting. Sway more and are more variable (not significant)." |  |
| Age differences in visual sensory integration  Teasdale, 1991 | 7; 64-78; 72.4(n.r.); 42.9% | 7; 18-23; 21.6(n.r.); 28.6% | - | Force platform, speaker, computer | Postural task | Successively open and close their eyes every 30s | | Maintain posture | 15min, 5 trials | "Results slowed that young and elderly subjects' sway dispersion increased when they were exposed to a reduced visual sensory condition (i.e., vision/no-vision transition). However, when exposed to augmented sensory condition (i.e., no-vision/vision transitions) young adults were able to adapt rapidly and reduced their sway dispersion whereas the elderly exhibited an increased sway dispersion." |  |
|  | | | | | | | | | | |  |
| ***Tests on the visual and somatosensory modalities*** | | | | | | | | | | |  |
| **Title, first author, year of publication** | **Study characteristics** | | | | | | | | | **Results on aging** |  |
|  | **Participants (number; range; mean(SD); percentage of men)** | | | **Material** | **Experiments** | | | | |  |  |
|  | **Older adults** | **Younger adults** | **Other groups** |  | **Tasks** | **Conditions** | | **Instructions** | **Time and repetition** |  |  |
| The impact of eye closure on somatosensory perception in the elderly  Brodoehl, 2015 | 16; 62–71; 66.9(5.2); 56.3% | 18; 21–28; 23(1.6); 44.4% | - | fMRI, darkened room, clinical neurostimulator, darkened googles | Detection task | Wave pulses administered to right index finger  A) Current perception threshold  1) Eyes open  2) Eyes closed  B) fMRI experiments  1) No tactile stimulation  2) Tactile stimulation | | A) Indicate when feel stimulus  B) Open and close their eyes every 27 s | 25 min | "Improved perception threshold in both groups when the eyes were closed, but the improvement was significantly less pronounced in the elderly. fMRI data revealed increased resting activity in the somatosensory cortex with closed eyes, and the stimulus-induced activity of the secondary somatosensory cortex decreased in the young but not in the elderly." |  |
| Perceptual processing strategies in the cross-modal transfer of form discrimination: A developmental study  Coté, 1981 | 20; 60-80; 68.4(n.r.); 50% | 20; 16-20; 18.6(n.r.); 50% | Children; 20; n.r.; 6 years 11 months  (n.r.); 50% | Black letters on white plastic coated cards, smooth square metal background mounted on wooden blocks, wooden box, special googles, screen | Tactual transfer task | A) Visual training  1) Partial vision (googles)  2) Whole vision  B) Tactual task | | A) Discriminate two visual stimuli (same or different)  B) Discriminate two tactual stimuli (same or different) | A) 48 trials  B) 48 trials | "There was no evidence of transfer for the children. Specific and nonspecific cross-modal transfer was found in the high school and elderly groups following partial vision training. Positive transfer was also found for the high schoolers in the whole vision condition. Whereas all age groups performed equally well on standard vs transformation comparisons, standard vs standard matches were highest in the high school subjects, lowest in the children, and intermediate in the elderly. " |  |
| Aging and the visual, haptic, and cross-modal perception of natural object shape  Norman, 2006 | Exp 1:  30; 63-82; 73.5(4.7); 50%  Exp 2:  30; 60-81; 70(5.8); 50% | Exp 1:  30; 18-31; 21.7(3.4); 50%  Exp 2:  10; 19-22; 20.8(1); 50% | - | 2 sets of plastic copies of 12 ordinary bell peppers | Perception task | A) Two sequentially presented objects  1) Haptic-haptic  2) Visual-visual  3) Haptic-visual  B) 12 visible objects and 12 objects known only by active touch | | A) Judge if the two objects were the same in 3D shape  B) Match 3D shape of haptic object with one of the 12 visible objects | 120 trials | "Quantitative effects of age were found in both experiments. The effect of age in experiment 1 was limited to cross-modal shape discrimination: there was no effect of age upon unimodal (ie within a single perceptual modality) shape discrimination. The effect of age in experiment 2 was eliminated when the older observers were either given an unlimited amount of time to perform the task or when the number of response alternatives was decreased." |  |
| Cross-modal functions in alcoholism and aging  Oscar-Berman, 1990 | 13; n.r.; 61(n.r.); 100% | 13; n.r.; 37(n.r.); 100% | Young alcoholics: 10; n.r.; 36(n.r.); 100%  Older alcoholics: 14; n.r.; 57(n.r.); 100%  Alcoholic Korsakoff patients: 5; n.r.; 64(n.r.); 100% | 60 3D objects mounted onto circular Plexiglas basis, stimulus tray, curtain coin dispenser, national semiconductor microprocessor, computer | Object recognition | A) Experiment 1  1)Visual to tactual recognition  2)Tactual to visual recognition  B) Experiment 2  1) Tactual discrimination  a) Texture relevant training  b) Shape relevant  2) Visual discrimination  a) Texture relevant training  b) Shape relevant training  3) Crossmodal problem: Texture relevant | | A) Match the visible object with one of two using touch or reverse  B) 1) Find the target object between two with touch  2) Find the target object between two with vision  3) Match the visible object with one of two using touch or reverse | A) 20 trials  B) 20 trials | "Results indicated that aging is associated with decline in tactual discrimination ability. Further, cross-modal functions appear to be compromised by alcoholic Korsakoff's disease’ and-to a lesser extent-by the combined effects of alcoholism and normal chronological aging." |  |
| Vision and touch in ageing: Crossmodal selective attention and visuotactile spatial interactions  Poliakoff, 2006a | 24; 76–92; 80.8(n.r.); n.r. | 24; 19–25; 21.7(n.r.); n.r. | Young-Old: 24; 65–72; 69.4(n.r.); n.r. | LEDs, 2 foam cubes with 2 LEDs each and 2 bone conduction vibrators each, foot pedals, headphones | Selective attention task | Participants held a foam cube in each hand  A) Posture conditions  1) Arms crossed  2) Arms uncrossed  B) Distractors in the other modality  1) No distractor  2) Congruent distractor, same side  3) Incongruent distractor, opposite side | | Indicate the elevation of the stimulus in the target modality (indicated before test) | 512 trials | "When attending to touch, the addition of visual distractors had a significantly larger effect on error rates in the older groups as compared to the Young group. In all three age groups, performance was impaired when the target and distractor were presented at incongruent as compared to congruent elevations. This congruency effect was modulated by the relative spatial location of the target and distractor in certain conditions for the Young and the Young-Old group. That is, participants in the two younger age groups found it harder to attend selectively to targets in one modality, when distractor stimuli came from the same side rather than from the opposite side. However, no significant spatial modulation was found in the Old-Old group. This suggests that ageing may also compromise spatial aspects of crossmodal selective attention." |  |
| Visuotactile temporal order judgments in ageing  Poliakoff, 2006b | 18; 67–84; 74.2(n.r.); 44.4% | 18; 19–25; 21.8(n.r.); 22.2% | - | LEDs, 2 foam cubes with 2 LEDs each and 2 bone conduction vibrators each, foot pedals, headphones | Temporal order judgment task | Participants held a foam cube in each hand, different SOA between stimuli presentations were used | | Determine whether the vibration or the visual input occurred first | 300 trials | "Temporal precision, as indexed by the just noticeable difference (JND), was better (i.e., JNDs were lower) when the stimuli were presented from different positions (M = 101 ms) rather than from the same position (M = 120 ms), as has been demonstrated previously. Additionally, older observers required more time (i.e., their JNDs were larger) to accurately perceive the temporal order (M = 131 ms) as compared to younger observers (M = 98 ms)." |  |
| Subjective straight-ahead during neck muscle vibration: Effects of ageing  Strupp, 1999 | 30; 20-81; 46.1(12.9); 60% | | | Half-spherical screen, experimental electromechanical physiotherapy vibrator, laser spot, head holder | Visual straight-ahead task | Participants seated in the center of a half spherical screen in total darkness  A) No stimulation  B) Dorsal muscles vibration | | Move a laser spot horizontally on the screen to the position they perceived as straight ahead | 14 x 20s stimulation | "They found a symmetrical increase of the vibration-induced displacement of the subjective visual straight-ahead with advancing age." |  |
| Aging o” sensorimotor processes: A systematic study in Fitts' task  Temprado, 2013 | 12; n.r.; 78(4); 50% | 9; n.r.; 25(2); 66.7% | - | Surface tablet, stylus, computer, custom software | Fitt’s task | Home-to-target aiming movement by sliding a hand-held non-marking stylus over the surface of a tablet, different distances and targets sizes | | Make home-to-target movements | 16 trials | "Results showed that older participants were always slower. However, in both age groups, movements were longer in distance manipulation, which resulted from a slowing of both acceleration times and deceleration times, while weight manipulation affected mainly deceleration times. In distance manipulation, equivalent age-related slowing ratios were observed for acceleration time and deceleration time (1.3). In weight manipulation, acceleration times of older participants were additively slower than those of young participants. Conversely, deceleration times presented a multiplicative slowing ratio of 1.3." |  |
|  | | | | | | | | | | | |
| ***Tests on other modalities*** | | | | | | | | | | | |
| **Title, first author, year of publication** | **Study characteristics** | | | | | | | | | **Results on aging** |  |
|  | **Participants (number; range; mean(SD); percentage of men)** | | | **Material** | **Experiments** | | | | |  |  |
|  | **Older adults** | **Younger adults** | **Other groups** |  | **Tasks** | **Conditions** | | **Instructions** | **Time and repetition** |  |  |
| The influence of age and surface compliance on changes in postural control and attention due to ankle neuromuscular fatigue  Bisson, 2014 | 13; n.r.; 65(4); 100% | 11; n.r.; 24(3); 100% | - | Dynamometer chair, force platform, block of dense foam, opaque ski googles, speakers | Postural task | Participants stand on a force platform blindfolded  A) Surface  1) Firm surface  2) Compliant surface  B) Fatigue condition  1) Baseline  2) Post fatigue trials  C) Dual task  1) No dual task  2) Choice reaction time task between two auditory stimuli | | Maintain posture | 16min | " Older adults are affected to a greater extent by muscle fatigue compared with young adults, but only when standing on a compliant surface. Only older men showed an increase in standing Choice Reaction Time with fatigue, and only when standing on the compliant surface." |  |
| The effect of ageing on multisensory integration for the control of movement timing  Elliott, 2011 | 15; 63–80; 72.6(n.r.); 33.3% | 15; 18–37; 27.7(n.r.); 60% | - | Piezo-electric auditory buzzer, solenoid-based tactile actuator, force sensitive resistor, headphones, data acquisition device | Control of movement timing | Metronome presentation:  A) Auditory  B) Tactile  C) Audio-tactile with temporal jitter to the auditory metronome  1) Temporal jitter: 0ms  2) 30ms  3) 60ms | | Tap the index finger in time to the metronome | 91 trials | "Older adults matched the performance of young adults when synchronizing to an isochronous auditory or tactile metronome. When the temporal regularity of the auditory metronome was reduced, older adults' performance was degraded to a greater extent than the young adults in both unimodal and bimodal conditions. However, proportionally both groups showed similar improvements in synchronization performance in bimodal conditions compared with the equivalent, auditory-only conditions." |  |
| Visual-vestibular stimulation interferes with information processing in young and older humans  Furman, 2003 | 20; 65-76; 69.3(3.2); 50% | 20; 20-30; 23.5(2.9); 50% | - | Rotational chair, rigid cylindrical enclosure | Visual-vestibular tasks while doing an information processing task | A) Visual-vestibular tasks  1) Vision only  2) Vestibular only, rotations on a rotational chair  3) Visuo-vestibular  B) Information processing tasks  1) Simple reaction time task  2) Disjunctive reaction time task  3) Forced-choice reaction time task | | A) Fixate a target  B) 1) Push button as soon as possible  2) Push button if target tone only  3) Push button in dominant hand if target tone, in non-dominant hand if non-target tone | 288 trials | "Results showed that older subjects had longer reaction times for all combinations of stimulus condition and reaction-time task compared with young subjects." |  |
| A model-based approach to attention and sensory integration in postural control of older adults Mahboobin, 2007 | 10; 61–85; 73(8); 40% | 10; 22–33; 25(3); 50% | - | Posture platform, earphones, computer with custom software | Postural task | Rotating posture platform during eyes-closed stance while performing an information processing task  A) No information processing task  B) Auditory choice reaction time  C) Auditory vigilance task | | A) Maintain posture  B) Maintain posture, press left or right button if hear a low or high pitch tone  C) Maintain posture, remember the number of low and high tone they heard | 9 trials | "Performing a concurrent Information Processing task had an overall effect on the time delay. Differences in the time delay of the postural control model were found for the older adults. The results suggest enhanced vulnerability of balance processes in older adults to interference from concurrent cognitive information processing tasks." |  |
| The effect of multisensory cues on attention in aging  Mahoney, 2011 | 18; n.r.; 76.4(7.9); n.r. | 18; n.r.; 19.2(2.7); n.r. | - | Constant voltage linear isolated programmable stimulator, electrodes, foot pedal, headphones, computer, screen | Detection task | A) Localization  1) Lateral  2) Bilateral  B) Modalities  1) Visual  2) Auditory  3) Somatosensory  4) Audiovisual  5) Audio-somatosensory  6) Visuo-somatosensory | | Respond as fast as possible to the stimuli | 232 trials | "Results revealed that reaction time to all multisensory pairings was significantly faster than those elicited to the constituent unisensory conditions across age groups. Both young and old participants responded the fastest to multisensory pairings containing somatosensory input. Compared to younger adults, older adults demonstrated a significantly greater reaction time benefit when processing concurrent visuo-somatosensory information. In terms of co-activation, older adults demonstrated a significant increase in the magnitude of visual-somatosensory co-activation (i.e., multisensory integration), while younger adults demonstrated a significant increase in the magnitude of auditory-visual and auditory-somatosensory co-activation." |  |
| Multisensory integration across the senses in young and old adults  Mahoney, 2012 | 18; n.r.; 76.4(7.9); 39% men | 18; n.r.; 19.2(2.7); 55% men | - | Constant voltage linear isolated programmable stimulator, electrodes, foot pedal, headphones, computer, screen | Forced-choice reaction time task | Rows of 5 block lines with arrowheads pointed leftward or rightward.  A) No cue  B) Sensory alerting cues, presented bilaterally  C) Sensory orienting cues presented at the location of the target stimulus  For B) and C)  1) Audiovisual  2) Audio-somatosensory  3) Visuo-somatosensory  4) Visual  5) Auditory  6) Somatosensory | | Make left or a right foot pedal presses in response to the direction of the central arrow | 232 trials | "Results revealed main effects for the executive attention and orienting networks, but not for the alerting network. Both old and young adults demonstrated significant orienting effects for audio-somatosensory, audiovisual, visuo-somatosensory cues. Younger adults demonstrated greater reaction time benefits for audio-somatosensory orienting cues whereas older adults demonstrated greater reaction time benefits for audiovisual orienting cues. Both groups demonstrated significant reaction time benefits for multisensory visuo-somatosensory orienting cues." |  |

n.r.= not reported
